# Extracellular matrix-derived mechanical force governs breast cancer cell stemness and quiescence transition through integrin-DDR signaling

**DOI:** 10.1038/s41392-023-01453-0

**Published:** 2023-06-28

**Authors:** Cong Li, Shi Qiu, Xiaohan Liu, Fengzhu Guo, Jingtong Zhai, Zhijun Li, Linghui Deng, Liming Ge, Haili Qian, Lu Yang, Binghe Xu

**Affiliations:** 1 Department of Medical Oncology, National Cancer Center/National Clinical Research Center for Cancer/Cancer Hospital, Chinese Academy of Medical Sciences and Peking Union Medical College, Beijing, 100021 China; 2 State Key Laboratory of Molecular Oncology, National Cancer Center/National Clinical Research Center for Cancer/Cancer Hospital, Chinese Academy of Medical Sciences and Peking Union Medical College, Beijing, 100021 China; 3 Department of Urology, Institute of Urology and National Clinical Research Center for Geriatrics, West China Hospital of Sichuan University, Chengdu, 610041 China; 4 National Clinical Research Center of Geriatrics, The Center of Gerontology and Geriatrics, West China Hospital, Sichuan University, Chengdu, 610065 China; 5 Institute of Oncology Research (IOR), Oncology Institute of Southern Switzerland (IOSI), Bellinzona, 6500 Switzerland; 6 Department of Histology and Embryology, Basic Medical College, China Medical University, Shenyang, Liaoning 110122 China; 7 Department of Pharmaceutical and Bioengineering, School of Chemical Engineering, Sichuan University, Chengdu, 610065 China

**Keywords:** Breast cancer, Cancer microenvironment

## Abstract

The extracellular matrix (ECM) serves as signals that regulate specific cell states in tumor tissues. Increasing evidence suggests that extracellular biomechanical force signals are critical in tumor progression. In this study, we aimed to explore the influence of ECM-derived biomechanical force on breast cancer cell status. Experiments were conducted using 3D collagen, fibrinogen, and Matrigel matrices to investigate the role of mechanical force in tumor development. Integrin-cytoskeleton-AIRE and DDR-STAT signals were examined using RNA sequencing and western blotting. Data from 1358 patients and 86 clinical specimens were used for ECM signature-prognosis analysis. Our findings revealed that ECM-derived mechanical force regulated tumor stemness and cell quiescence in breast cancer cells. A mechanical force of ~45 Pa derived from the extracellular substrate activated integrin β1/3 receptors, stimulating stem cell signaling pathways through the cytoskeleton/AIRE axis and promoting tumorigenic potential and stem-like phenotypes. However, excessive mechanical force (450 Pa) could drive stem-like cancer cells into a quiescent state, with the removal of mechanical forces leading to vigorous proliferation in quiescent cancer stem cells. Mechanical force facilitated cell cycle arrest to induce quiescence, dependent on DDR2/STAT1/P27 signaling. Therefore, ECM-derived mechanical force governs breast cancer cell status and proliferative characteristics through stiffness alterations. We further established an ECM signature based on the fibrinogen/fibronectin/vitronectin/elastin axis, which efficiently predicts patient prognosis in breast cancer. Our findings highlight the vital role of ECM-derived mechanical force in governing breast cancer cell stemness/quiescence transition and suggest the novel use of ECM signature in predicting the clinical prognosis of breast cancer.

## Introduction

Extracellular matrix (ECM) is a non-cellular component of the tumor microenvironment and is secreted by different types of cells to provide biophysical and biochemical support.^1^ It is composed of proteoglycans and glycoproteins that form complex extracellular protein networks, which create supramolecular aggregates such as fibrils and lamellar networks. During cancer development, the ECM undergoes persistent remodeling characterized by collagen degradation, deposition, and cross-linking.^2^ In addition to biochemical alterations, the biophysical parameters of ECM also change dynamically, including topography, stiffness, and molecular density. Previous studies have suggested that dynamic ECM remodeling plays a crucial role in tumorigenesis and tumor progression.^3^ The diverse composition of ECM components, including fibrous proteins (collagen), glycoproteins (fibronectin and fibrinogen), and proteoglycans, also participates in tumor progression via multiple molecular mechanisms.^4–6^ However, the specific role of the ECM in remodeling tumor behavior remains controversial. For example, while collagen has been previously considered a passive barrier for cancer cell growth, recent researches suggest that alterations in collagen deposition can either promote or inhibit tumor malignancy.^7–9^ Thus, further investigation is necessary to elucidate the interactive functions and synergistic effects of different ECM components.

Accumulating evidence suggests that a suitable ECM composition could facilitate the development of cancer stem cells (CSCs), leading to tumor initiation and relapse.^9^ ECM components such as fibronectin and collagen, regulate CSCs self-maintenance, including proliferation, quiescence, differentiation and apoptosis, through intricate mechanisms. For example, in breast cancer, collagen I and collagen VI could induce the expression of CSCs markers, while collagen IV and vitronectin promote CSCs differentiation, leading to a decreased CD44+ /CD24- CSCs population.^10^ ECM compounds such as fibronectin and hyaluronic acid were also reported to promote the enrichment of CD44+ glioblastoma stem cells.^11,12^ Increasing investigations provide evidence that ECM composition dynamically regulates CSCs development and dormancy, which determines drug resistance and tumor relapse occurrence. For instance, collagen IV and VI have been reported to contribute to the dormancy and reactivation of hematopoietic stem cells.^13^ However, the relationship between ECM compounds and CSCs dormancy remains unclear. Previous studies showed that dormant cells lose fibronectin connection and integrin activation in head and neck squamous cell cancer models,^14^ while recent studies indicated that dormant cells can organize fibronectin to maintain quiescence status in breast cancer cells.^15^ Besides, compelling research supports a direct role for collagen I in awakening dormant breast cell line D2.0R,^16^ while another study suggested that a 3D collagen I culture model could induce the dormancy of bladder cancer cells.^17^ These fundings suggest that ECM components may play multiple roles in regulating CSCs maintenance and dormancy. Recently, the importance of ECM stiffness as a crucial regulator of CSCs functions and metabolic processes in multiple tumor types has been increasingly recognized. This regulation is tissue-specific, with the tissue origin of cancer cells determining the optimal stiffness range for tumor growth. Moreover, increased matrix stiffness and alignment have been identified as hallmarks in many cancers, such as breast cancer, pancreatic and colorectal cancers.^18,19^ Recent research indicates that fibrin stiffness can induce the dormancy of tumor repopulation cells, suggesting the role for mechanical force in regulating cancer cell stemness and quiescence transition.^20^ However, the mechanisms underlying the physical properties and mechanical force that regulate CSCs status remain to be elucidated.

Understanding how ECM composition and mechanical properties regulate cancer cells function is a rapidly growing field. ECM components are known to directly bind to cellular transmembrane receptors and transduce biochemical signaling to facilitate the epithelial-mesenchymal transition and promote tumor progression, such as the focal adhesion kinase/Src and protein kinase A/Smad-1 pathways.^21,22^ In addition, ECM fibers can transmit biomechanical signals to affect cellular functions.^23^ Membrane receptors and channels connect the ECM and intracellular signal pathways. Receptors including integrins and CD44 can sense ECM composition and mechanical forces. Meanwhile, mechanical forces can activate cadherin receptors and mechanical ion channels by stretching plasma membrane. Significantly, integrins are activated under extracellular mechanical forces, cytoplasmic focal adhesion proteins connect to actomyosin (myosin II and filamentous actin), leading to the accumulation of cytoskeletal prestress.^24^ Cytoskeletal prestress can then facilitate long-range cytoplasmic mechanical transduction, enabling direct chromatin stretching and rapid gene expression.^24^ Despite the critical and dynamic role of biomechanical force in determining tumor biological characteristics, the underlying mechanisms how extracellular prestress modulates cytoplasmic or nuclear proteins to influence tumor progression remain poorly understood.

In the present study, we aimed to investigate the impact of ECM-derived biomechanical force on breast cancer cell status and explore the underlying molecular mechanisms. Our results demonstrated that ECM-derived biomechanical force governs breast cancer cells stemness and quiescence transition. We elucidated the molecular mechanism of how biomechanical force induces tumor progression, showing that it upregulates cancer stemness through integrin-cytoskeletal prestress-AIRE signals, while mediating the quiescence of stem like tumor cells through DDR/STAT1/P27 signaling. Our findings highlight the role of biomechanical signaling in ECM-induced tumor progression in breast cancer, providing innovative perspectives in both biomechanics and oncology.

## Results

### ECM-induced biomechanical force promotes breast tumor stemness

**Fig. 1:**
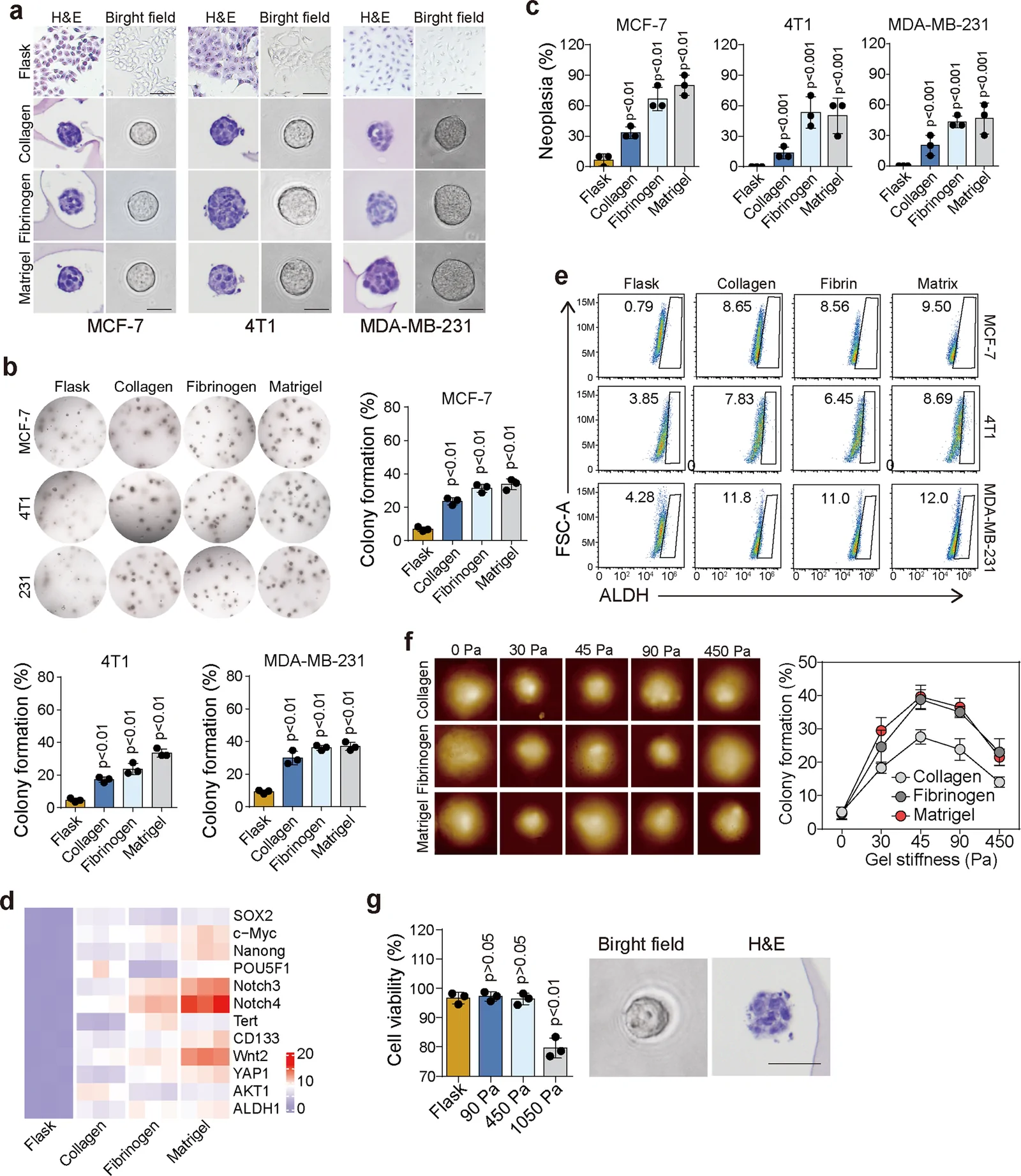
The ECM-induced biomechanical force promoted breast tumor stemness. **a** Representative images and H&E staining images of MCF-7, 4T1 and MDA-MB-231 cells seeded in a flask system and different 3D gels (collagen, fibrinogen, and Matrigel) for 3 days. The scale bar was 50 μm. **b**, **c** MCF-7, 4T1 and MDA-MB-231 cells were cultured in a flask system or 3D gels (collagen, fibrinogen, and Matrigel) for 3 days. In vitro colony formation (**b**) and in vivo tumor formation assay (*n* = 10) (**c**) were performed. **d** Heatmap of stemness-associated genes (*SOX2, c-Myc, Nanog, POU5F1, Notch3, Notch4, Tert, CD133, Wnt2, YAP1, AKT1*, and *ALDH1*) expression in MCF-7 cells cultured in flask and different 3D gels (collagen, fibrinogen, and Matrigel) for 3 days, determined using qPCR. **e** MCF-7, 4T1 and MDA-MB-231 cells were cultured in a flask system or 3D gels (collagen, fibrinogen, and Matrigel) for 3 days. ALDH1^+^ cell subpopulations were determined by flow cytometry. **f** MCF-7 cells were seeded in different 3D gels (collagen, fibrinogen, and Matrigel) with different stiffness (0, 30, 45, 90, and 450 Pa) for 3 days. Following this, the in vitro colony formation assay was performed. Representative images of tumor cells during atomic force microscopy analysis are shown. **g** Viability of MCF-7 cells seeded in a flask or 3D Matrigel (90, 450, and 1050 Pa). Representative image and H&E staining of MCF-7 cells seeded in 3D Matrigel (1050 Pa, 3 days) are shown. The scale bar is 50 μm. Three independent experiments were performed. Data are represented as mean ± SEM. *P* < 0.05, statistical significance

To clarify the role of the ECM or, more specifically, the role of the ECM-induced biomechanical force, in driving stem-like phenotypes within tumor cells, we first seeded breast cancer cells 4T1, MCF-7 and MDA-MB-231 in different 3D gels (collagen, fibrinogen, and Matrigel), as previously described. Subsequently, we determined the tumorigenic potential using in vitro colony formation and in vivo subcutaneous neoplasia assays. The 4T1, MCF-7, and MDA-MB-231 breast cancer cells exhibited a rounded morphology (Fig. 1a) and enhanced tumorigenic potential (Fig. 1b, c) when cultured in 3D collagen, fibrinogen, or Matrigel gel. Concordantly, transcriptome analysis revealed that the tumor cells cultured in the 3D system exhibited pronounced upregulation of stemness-associated genes (Fig. 1d and Supplementary Fig. 1a) and breast cancer stem cell markers (ALDH1^+^, Fig. 1e). However, soluble collagen, fibrinogen, or Matrigel compounds (collagen, laminin, and fibronectin, respectively) showed limited influence on tumorigenic potential (Supplementary Fig. 1b, c) and stem cell marker expression (Supplementary Fig. 1d, e) in MCF-7 cells. These results suggest that ECM compounds may regulate breast tumor stemness through ECM-induced biomechanical force signals instead of chemical signals.

To better understand the role of ECM-induced biomechanical force on tumor stemness, tumor cells were seeded in ECM gels with multifold stiffness by adjusting the substrate concentration. And the colony formation potential was subsequently assayed. The biomechanical force induced by ECM was measured using atomic force microscopy. Notably, MCF-7 or 4T1 cells cultured in 45-Pa gels displayed an enhanced potential in colony formation, whereas excessive or exiguous biomechanical force weakened the tumorigenic potential (Fig. 1f and Supplementary Fig. 1f). Furthermore, excessive biomechanical force (>1000 Pa) increased apoptosis and induced structural damage in tumor cells (Fig. 1g). Collectively, these results suggest that ECM-induced biomechanical force (30–450 Pa) could mediate stem-like phenotypes and promote tumorigenic potential in breast cancer cells.

### ECM compounds bind to integrins to transduce biomechanical force signals

**Fig. 2:**
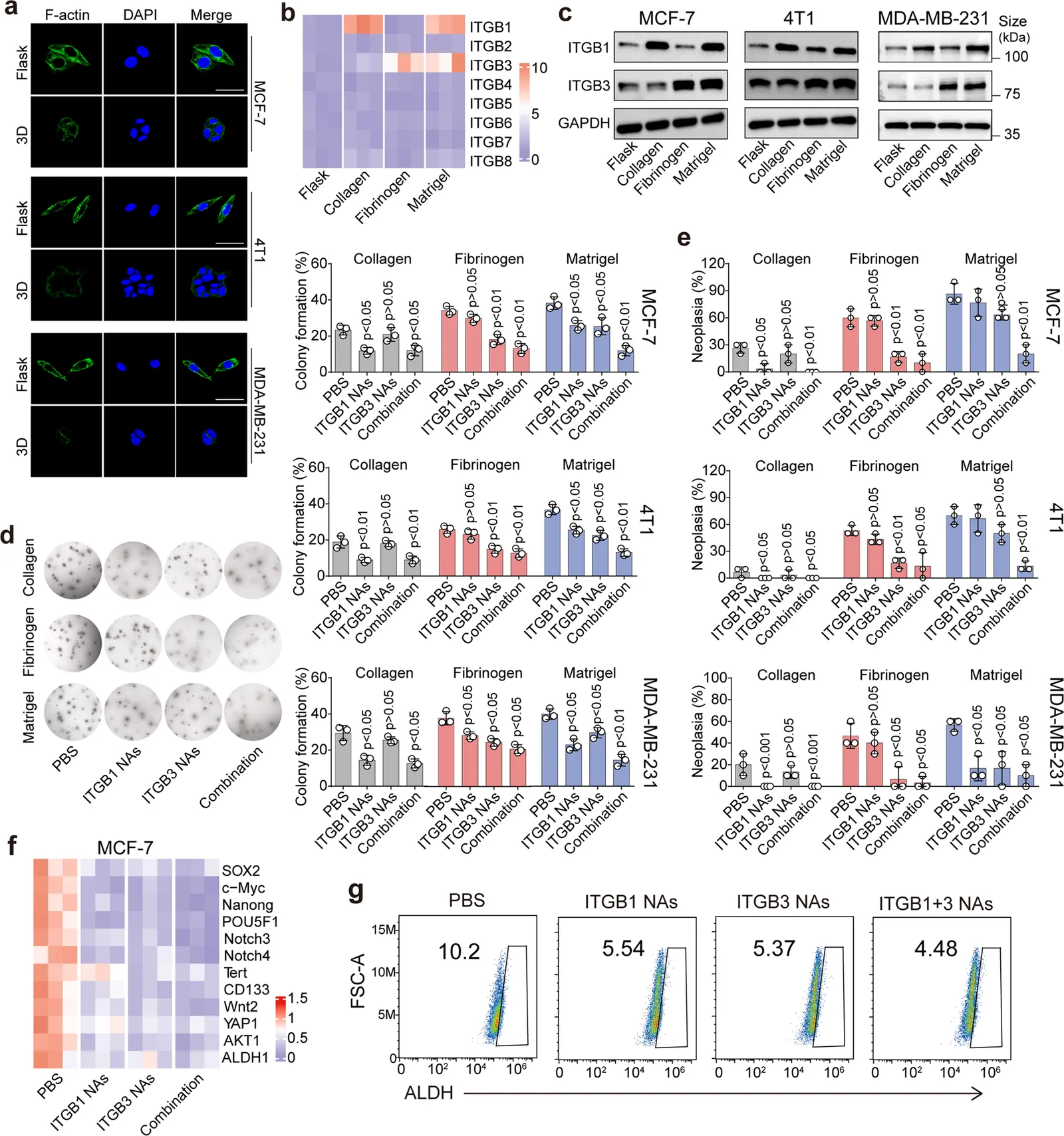
ECM compounds bind to integrins to transduce biomechanical force signals. **a** Immunostaining of F-actin in the MCF-7, 4T1 and MDA-MB-231 cells cultured in a flask and 3D Matrigel for 3 days. The scale bar is 20 μm. **b** Heatmap of integrin β1 ~ 8 expression in MCF-7 cells cultured in a flask and different 3D gels (collagen, fibrinogen, and Matrigel) for 3 days. **c** Western blotting of integrin β1 and β3 in MCF-7, 4T1 and MDA-MB-231 cells cultured in a flask and different 3D gels (collagen, fibrinogen, and Matrigel) for 3 days. **d**, **e** in vitro colony formation (**d**) and in vivo tumor formation (*n* = 10) (**e**) assays for MCF-7/4T1/MDA-MB-231 cells seeded in different 3D gels (collagen, fibrinogen, and Matrigel) and treated with PBS and integrin β1- and β3-neutralizing antibodies, respectively. **f** Heatmap of stemness-associated gene (*SOX2, c-Myc, Nanog, POU5F1, Notch3, Notch4, Tert, CD133, Wnt2, YAP1, AKT1*, and *ALDH1*) expression in MCF-7 cells (3D Matrigel culture) treated with PBS and integrin β1- and β3-neutralizing antibodies. **g** ALDH1^+^ cell subpopulations were determined in MCF-7 cells (3D Matrigel culture) treated with PBS and integrin β1- and β3-neutralizing antibodies. Three independent experiments were performed. Data are represented as mean ± SEM. *P* < 0.05, statistical significance

ECM-induced biomechanical force is the major mediator of tumor stemness; therefore, breast cancer cells seeded in different gels under the same biomechanical force should exhibit similar tumorigenic potential. However, as shown in Figs. 1f and S1f, MCF-7 and 4T1 cells seeded in Matrigel (45 Pa) exhibited enhanced tumorigenic potential compared with cells seeded in collagen (45 Pa) or fibrinogen (45 Pa) gel. As previously reported, extracellular biomechanical force modified the conformation of integrins in force signaling receptors and thus modulated integrin activation, strengthening downstream stem signaling activation.^25^ Different integrin molecules bind to diverse components of the ECM (such as integrin β1-collagen I, and integrin β3-fibronectin), resulting in the upregulation of stem-associated genes in tumor cells.^26^ Therefore, we speculate that compounds in the ECM bind to different biomechanical force receptor-integrins and transduce force signals via the integrin-cytoskeleton axis to regulate downstream stem signaling pathways. We first observed cytoskeletal deformation in MCF-7, 4T1, and MDA-MB-231 cells cultured in the 3D gels (Fig. 2a). To confirm our hypothesis, the mRNA levels of integrins (β1–8) were measured in flask- and 3D gel-cultured MCF-7 cells. Collagen-cultured cells showed an increased integrin β1 expression; fibrinogen-cultured cells exhibited an increased integrin β3 expression, and Matrigel (containing collagen and fibronectin)-cultured cells demonstrated an increase in integrin β1 and 3 expression (Fig. 2b).

The elevated expression of integrins was examined at the protein level in MCF-7, 4T1, and MDA-MB-231 cells (Fig. 2c). In addition, a limited influence was observed in MCF-7 cells cultured with soluble Matrigel (Supplementary Fig. 2a). Subsequently, the MCF-7, 4T1, and MDA-MB-231 cells were incubated with integrin β1- or β3-neutralizing antibodies during 3D culture for 3 days. Integrin β1- and β3-neutralizing antibodies suppressed the tumorigenic potential induced by collagen and fibrinogen gels, respectively. The combination of integrin β1 and 3 antibodies inhibited colony formation and tumorigenic potential in MCF-7, 4T1, MDA-MB-231 cells in the Matrigel culture system (Fig. 2d, e). The influence of neutralizing antibodies on colony formation was limited in flask-cultured tumor cells (Supplementary Fig. 2b). Moreover, the blockade of integrin signaling suppressed the upregulation of stemness-associated genes and stem cell markers in cells cultured with 3D Matrigel (Fig. 2f, g). These results suggest that ECM compounds target specific integrins to mediate the transduction of biomechanical force signals.

Subsequently, we explored the downstream molecules of integrin signaling. We observed that mRNA levels of *YAP1* and *Notch3/4* were upregulated in 3D cultured tumor cells. Accordingly, increased nucleolar YAP1 and Notch3/4 protein levels were observed in the 3D cultured group (Supplementary Fig. 2c). Additionally, the blockade of YAP1 and Notch signaling by YAP-TEAD-IN-1 and Notch inhibitor 1 suppressed the colony formation induced by 3D gels (Supplementary Fig. 2d), indicating that integrins regulate breast cancer cell behaviors through YAP and Notch signals. Collectively, these results suggest the role of integrins in biomechanical force signal transduction, and the deficiency of specific ECM compounds/integrins can lead to the inefficiency of biomechanical force signals.

### Integrin-cytoskeleton-AIRE signals are crucial for stem gene upregulation

**Fig. 3:**
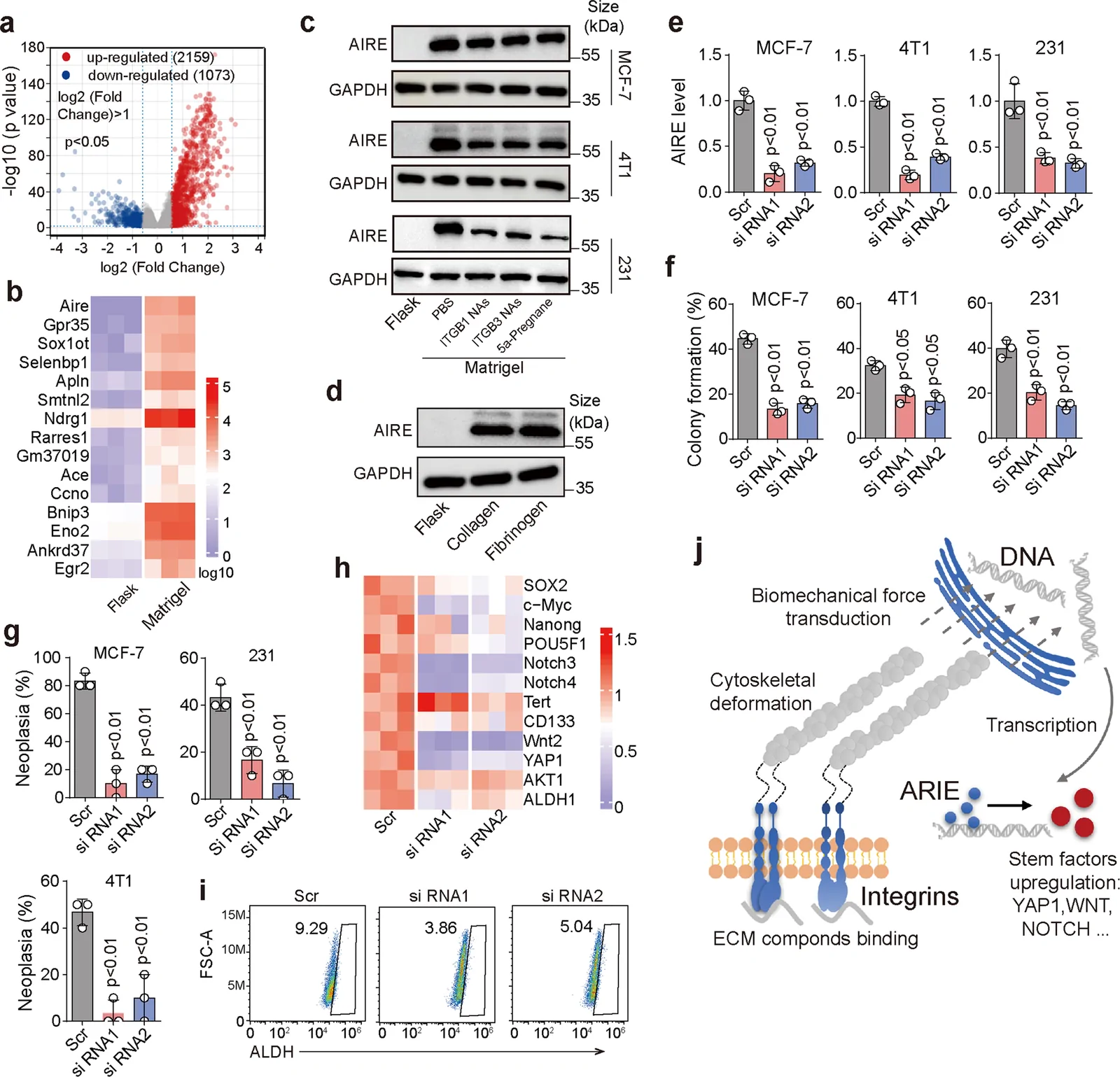
Integrin-cytoskeleton-AIRE signals are crucial for stemness gene upregulation. **a** Volcano plots showing the differentially expressed genes in 4T1 cells cultured in a flask and 3D Matrigel for 3 days. **b** Heatmap of top 15 upregulated genes in 3D Matrigel-cultured 4T1 cells in comparison with that of cells cultured in a flask. **c** Western blotting for AIRE in MCF-7, 4T1 and MDA-MB-231 cells cultured in a flask or 3D Matrigel (treated with PBS, integrin β1/3-neutralizing antibodies, or 5a-Pregnane-3,20-dione). **d** Western blotting for AIRE in MCF-7 cells cultured in a flask or 3D collagen/fibrinogen gels for 3 days. **e** AIRE expression at the mRNA level in 3D Matrigel-cultured MCF-7/4T1/MDA-MB-231 cells, treated with scramble or AIRE siRNA. **f**, **g** in vitro colony formation potential (**f**) and in vivo tumor formation (*n* = 10)(**g**) potential of 3D Matrigel-cultured MCF-7/4T1/MDA-MB-231 cells treated with scramble or AIRE siRNA. **h** Heatmap of stemness-associated gene (*SOX2, c-Myc, Nanog, POU5F1, Notch3, Notc4, Tert, CD133, Wnt2, YAP1, AKT1*, and *ALDH1*) expression in 3D Matrigel cultured MCF-7 cells treated with scramble or AIRE siRNA. **i** ALDH1^+^ cell subpopulations were determined in MCF-7 cells (3D Matrigel culture) treated with scramble or AIRE siRNA. **j** Schematic representation of the integrin-cytoskeleton-AIRE signals in breast cancer cells. Three independent experiments were performed. Data are represented as mean ± SEM. *P* < 0.05, statistical significance

RNA sequence analysis of 4T1 cells cultured in a flask or Matrigel was performed to investigate the mechanism underlying the changes in stemness. Differentially expressed genes were analyzed, and the top 15 upregulated genes are listed in Fig. 3a, b. Notably, *AIRE*, an autoimmune regulator, was considerably upregulated in cells cultured in 3D Matrigel. This protein is primarily active in the thymus and plays a vital role in immune system functions.^27,28^ However, the role of AIRE in tumor cells has rarely been reported. Therefore, we further examined AIRE expression in MCF-7, 4T1, and MDA-MB-231 cells and observed an increased AIRE protein level in 3D Matrigel-cultured cells. Furthermore, integrin β1- and β3-neutralizing antibodies or 5a-Pregnane-3,20-dione (cytoskeleton inhibitor, depolymerizing actin) suppressed AIRE upregulation in Matrigel cultured cells (Fig. 3c). A limited influence was observed in soluble Matrigel-treated MCF-7 cells (Supplementary Fig. 3a), indicating that ECM-induced biomechanical force activated AIRE signaling through the integrin/cytoskeleton axis. Enhanced expression of AIRE was observed in MCF-7 cells seeded in fibrinogen or collagen gels (Fig. 3d).

Subsequently, we used siRNA to silence AIRE in cells cultured in specific gels (Fig. 3e) and determined their tumorigenic potential and stemness-associated gene expression. Concordantly, *AIRE* silencing suppressed colony formation (Fig. 3f), tumorigenesis (Fig. 3g), and upregulation of stemness-associated genes and breast cancer stem cell markers (Fig. 3h, i). We also overexpressed AIRE in MCF-7 cells (Supplementary Fig. 3b). Enhanced colony formation capability (Supplementary Fig. 3c) and upregulated stemness-associated gene expression (Supplementary Fig. 3d) were observed in AIRE-overexpressed tumor cells. These results suggest that ECM-induced biomechanical force upregulates integrin-cytoskeleton-AIRE signaling to promote the stem-like phenotype in breast cancer cells (Fig. 3j).

### ECM-induced biomechanical force drives stem-like tumor cell quiescence

**Fig. 4:**
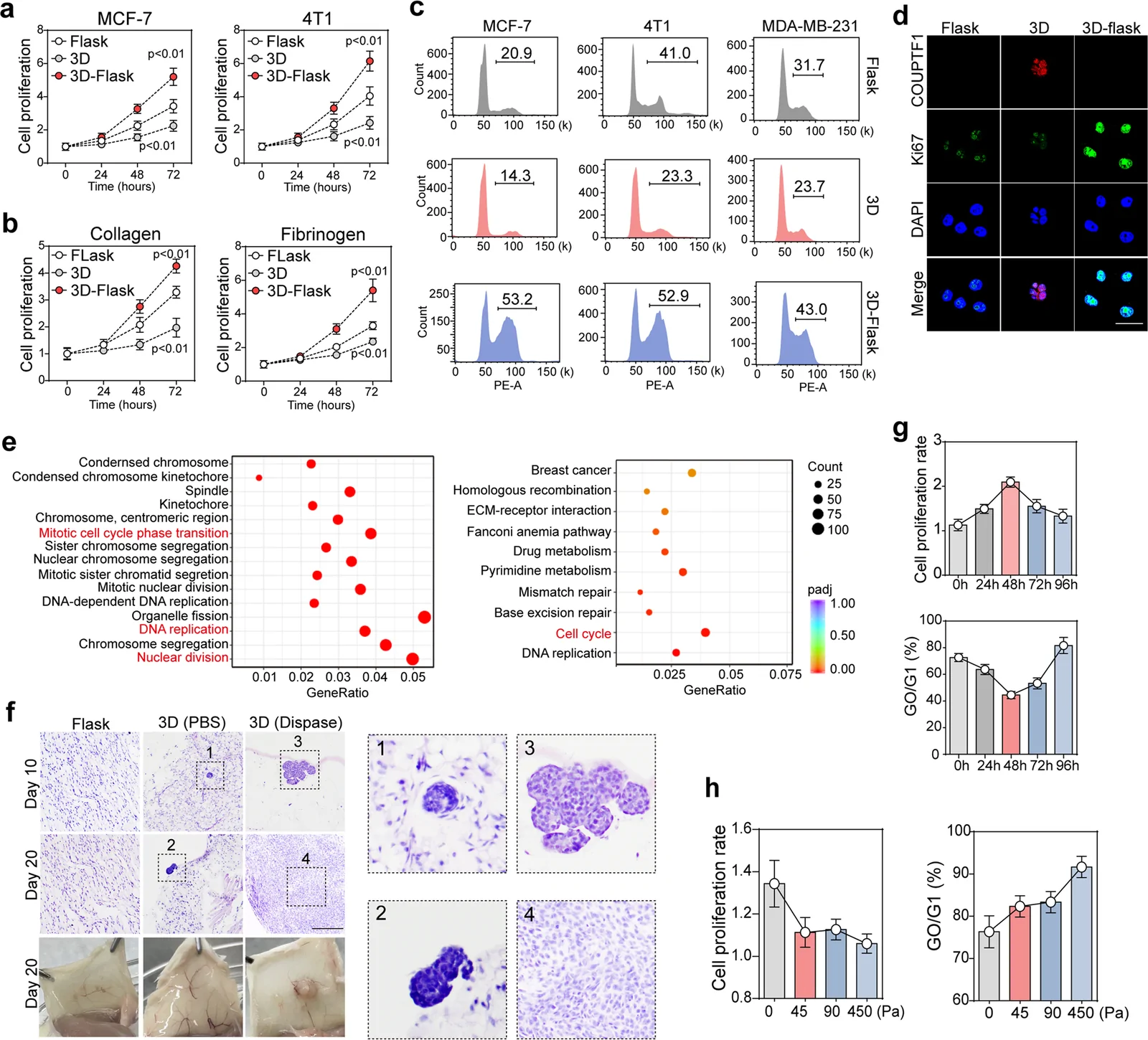
ECM-induced biomechanical force drives stem cell-like tumor cell quiescence. **a** MCF-7/4T1 cells were seeded in a flask and 3D Matrigel, following which the cells were harvested for the cell proliferation assay in a 96-well plate (flask and 3D-flask groups). Some of the 3D-cultured MCF-7/4T1 cells were re-seeded in 3D Matrigel and subjected to cell proliferation determination at the same time points (3D group). **b** Proliferation of MCF-7 cells cultured in a flask, 3D-flask, and 3D gel (3D collagen and fibrinogen culture system). **c** Cell cycle analysis of MCF-7/4T1/MDA-MB-231 cells cultured in a flask, 3D gels, and 3D-flask. **d** Immunostaining for Ki67 and CoupTF1 in the flask, 3D gel, and 3D-flask groups. The scale bar is 20 μm. **e** GO and KEGG enrichment analysis of differentially expressed genes in the 4T1 cells cultured in the flask and 3D culture system, with a significance threshold of *p*-value < 0.05. **f** 1 × 10^3^ 4T1 cells were encapsulated in a 450-Pa 3D Matrigel (or not) and subcutaneously implanted into mice. On days 3 and 5, the mice were treated with PBS or dispase administered via subcutaneous injection. H&E staining of the hypodermis in each group was performed on days 10 and 20 (*n* = 10). The scale bar was 500 μm. **g** MCF-7 cells were cultured in the 3D Matrigel for 3 days and isolated for flask culture. After 0, 24, 48, 72, and 96 h of flask culture, cell proliferation or cycle was examined. **h** MCF-7 cells were seeded in 45-, 90-, and 450-Pa Matrigel for 3 days. Cell cycle and proliferation (in Matrigel) were examined. Three independent experiments were performed. Data are represented as mean ± SEM. *P* < 0.05, statistical significance

As we observed that ECM-induced biomechanical force promoted tumor stemness, we contemplated that 3D gels facilitated breast cancer cell proliferation to promote tumor growth. However, tumor cells seeded in 3D gels exhibited weakened cell proliferation compared to flask-cultured cells. Notably, cell amplification increased remarkably in tumor cells isolated from 3D gels and seeded in flasks (Fig. 4a). Similar results were observed for MCF-7 cells cultured in 3D collagen and fibrinogen gels (Fig. 4b). In addition, cell cycle analysis showed that 3D gel-cultured 4T1, MDA-MB-231, and MCF-7 cells showed G0/G1 arrest, displaying a quiescence-like status. However, tumor cells isolated from 3D gels and seeded in a flask for 24 h exhibited reduced G0/G1 arrest compared with those isolated from flask culture or 3D Matrigel (Fig. 4c). These findings were consistent with the cell proliferative characteristics shown in Fig. 4a. However, the cell cycle of MCF-7 cells treated with soluble Matrigel was unchanged in comparison with the PBS group (Supplementary Fig. 4a).

Immunostaining results indicated that MCF-7 cells seeded in Matrigel showed elevated expression of CoupTF1 (a cell quiescence marker). In contrast, gel-to-flask-cultured MCF-7 cells showed enhanced Ki67 (a cell proliferation marker) (Fig. 4d). Moreover, transcriptomic analysis on flask/3D Matrigel-cultured 4T1 cells suggested that the 3D Matrigel culture directly modulated the cell cycle in breast cancer cells (Fig. 4e). These results indicate that the ECM-induced biomechanical force drives tumor cells to a dormancy-like status. The removal of ECM components drives palinesthesia, resulting in vigorous proliferation.

We further validated the role of biomechanical force in governing cell quiescence/palinesthesia in vivo, Matrigel-encapsulating or flask-cultured 1 × 10^3^ 4T1 or 5 × 10^3^ MCF-7/MDA-MB-231 cells were encapsulated in a 450-Pa 3D Matrigel (or not) and subcutaneously implanted into mice. On days 3 and 5, the mice were treated with PBS or dispase (for Matrigel degradation) administered via subcutaneous injection. Notably, no tumor formation was observed in mice injected with flask-cultured or Matrigel-encapsulating cells. However, dispase treatment caused macroscopic neoplasia in mice injected with gel-encapsulating cells. Subcutaneous tissue staining indicated that mice injected with Matrigel-encapsulating cells showed the formation of tiny tumor nests (1–1000 cells), which showed limited growth from days 10–20. In contrast, no tumor nests were found in mice injected with flask-cultured cells (Fig. 4f and Supplementary Fig. 4b). The results suggest that the 450-Pa 3D Matrigel may mediate tumor cell quiescence and stem-like phenotypes, and the degradation of ECM components by dispase results in cell palinesthesia and tumor formation, which aligns with our in vitro results.

The aberrant proliferative characteristics and reduced G0/G1 arrest in gel-to-flask cultured cells lasted >72 h (Fig. 4g). Additionally, tumor cells seeded in gels with greater stiffness exhibited enhanced G0/G1 arrest and suppressed proliferative characteristics (Fig. 4h). These results indicate that excessive ECM-induced biomechanical force may drive stem-like tumor cells to a dormancy-like status and that the removal of the ECM may induce cell palinesthesia and a vigorous proliferative status, thus promoting tumor formation and growth.

### Biomechanical force promotes tumor cell quiescence through DDR2 signaling

**Fig. 5:**
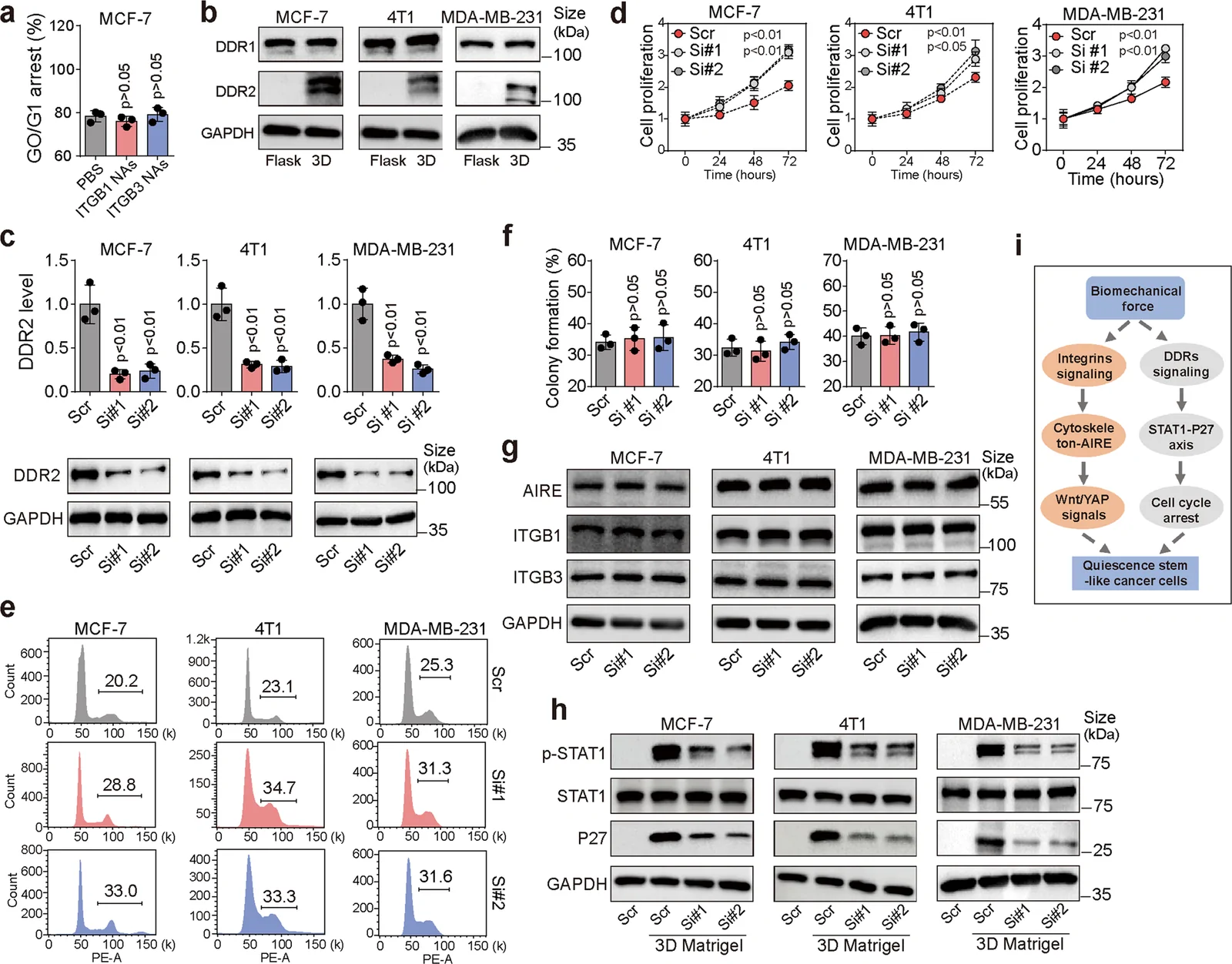
The biomechanical force promoted tumor cell quiescence through DDR2 signaling. **a** 3D Matrigel-cultured MCF-7 cells were treated with PBS and integrin β1/3-neutralizing antibodies. Following this, the cell cycle was determined. **b** Western blotting for DDR1 and DDR2 in MCF-7/4T1/MDA-MB-231 cells cultured in a flask or 3D Matrigel. **c** DDR2 expression at the mRNA and protein level in 3D Matrigel-cultured MCF-7/4T1/MDA-MB-231 cells treated with scramble or DDR2 siRNA. **d** Proliferation of 3D Matrigel-cultured MCF-7/4T1/MDA-MB-231 cells treated with scramble or DDR2 siRNA (in 3D Matrigel). **e** Cell cycle of 3D Matrigel-cultured MCF-7/4T1/MDA-MB-231 cells treated with scramble or DDR2 siRNA. **f** In vitro colony formation of MCF-7/4T1/MDA-MB-231 cells treated with scramble or DDR2 siRNA. **g** Western blotting of integrin β1, integrin β3, and AIRE in 3D Matrigel-cultured MCF-7/4T1/MDA-MB-231 cells treated with scramble or DDR2 siRNA. **h** Western blotting of phosphorylated STAT1, total STAT1, and P27 in flask/3D Matrigel-cultured MCF-7/4T1/MDA-MB-231 cells treated with scramble or DDR2 siRNA. **i** Schematic diagram of biomechanical force regulating breast cancer cell behaviors through DDRs and integrins signals. Three independent experiments were performed. Data are represented as mean ± SEM. *P* < 0.05, statistical significance

We explored the mechanism underlying biomechanical force-induced cell quiescence. Integrin receptors play a crucial physiological role in cell adhesion and reportedly promote cancer cell growth and migration; however, the blockade of integrin β1 or β3 signaling did not affect the cell cycle of Matrigel-cultured MCF-7 cells (Fig. 5a), which indicates that ECM-induced biomechanical force drives cell quiescence in an integrin-independent manner.

Compelling findings have suggested that tumor-derived type III collagen can affect the cell cycle and mediate tumor cell dormancy through DDRs.^29^ Thus, we examined the protein expression of discoidin domain receptor 1 (DDR1) and DDR2 in MCF-7, 4T1, and MDA-MB-231 cells cultured in 3D gels. The DDR2 protein level increased in cells cultured in Matrigel (Fig. 5b). To determine the role of DDR2, MCF-7, 4T1, and MDA-MB-231 cells were treated with DDR2-targeted siRNA (Fig. 5c). Intriguingly, reduced G0/G1 arrest and enhanced proliferation were observed in DDR2-silenced cells (Fig. 5d, e). However, the silencing of DDR2 did not affect the tumorigenic potential (Fig. 5f) or integrin-AIRE axis expression of the cells (Fig. 5g). These results indicate that ECM-induced biomechanical force promotes quiescence through DDR2 signaling and stem-like phenotype activation via integrin signaling.

Subsequently, we examined the expression of major cell cycle-associated proteins, P21, P27, P57, and STAT1. We observed that P27 was upregulated in 3D Matrigel-cultured MCF-7 cells (Supplementary Fig. 5a). The expression of phosphorylated STAT1 and P27 was elevated in Matrigel-cultured MCF-7, 4T1, and MDA-MB-231 cells, and the silencing of DDR2 suppressed STAT1/P27 upregulation induced by 3D culture (Fig. 5h). The expression of DDR2/STAT1/P27 was unaltered in MCF-7 cells treated with soluble Matrigel (Supplementary Fig. 5b). To further explore the role of DDR2 in vivo, we overexpressed DDR2 in MCF-7 cells. Consistently, weakened proliferative characteristics, enhanced cell cycle arrest, and activation of STAT1/P27 signal were found in DDR2 overexpressed MCF-7 cells in vitro (Supplementary Fig. 5c–e) and in vivo (Supplementary Fig. 5f–h). Collectively, these results suggest that ECM-induced biomechanical force promotes tumor cell quiescence through DDR2/STAT1 signaling (Fig. 5i).

### Novel ECM signature predicts clinical outcomes in patients with breast cancer

**Fig. 6:**
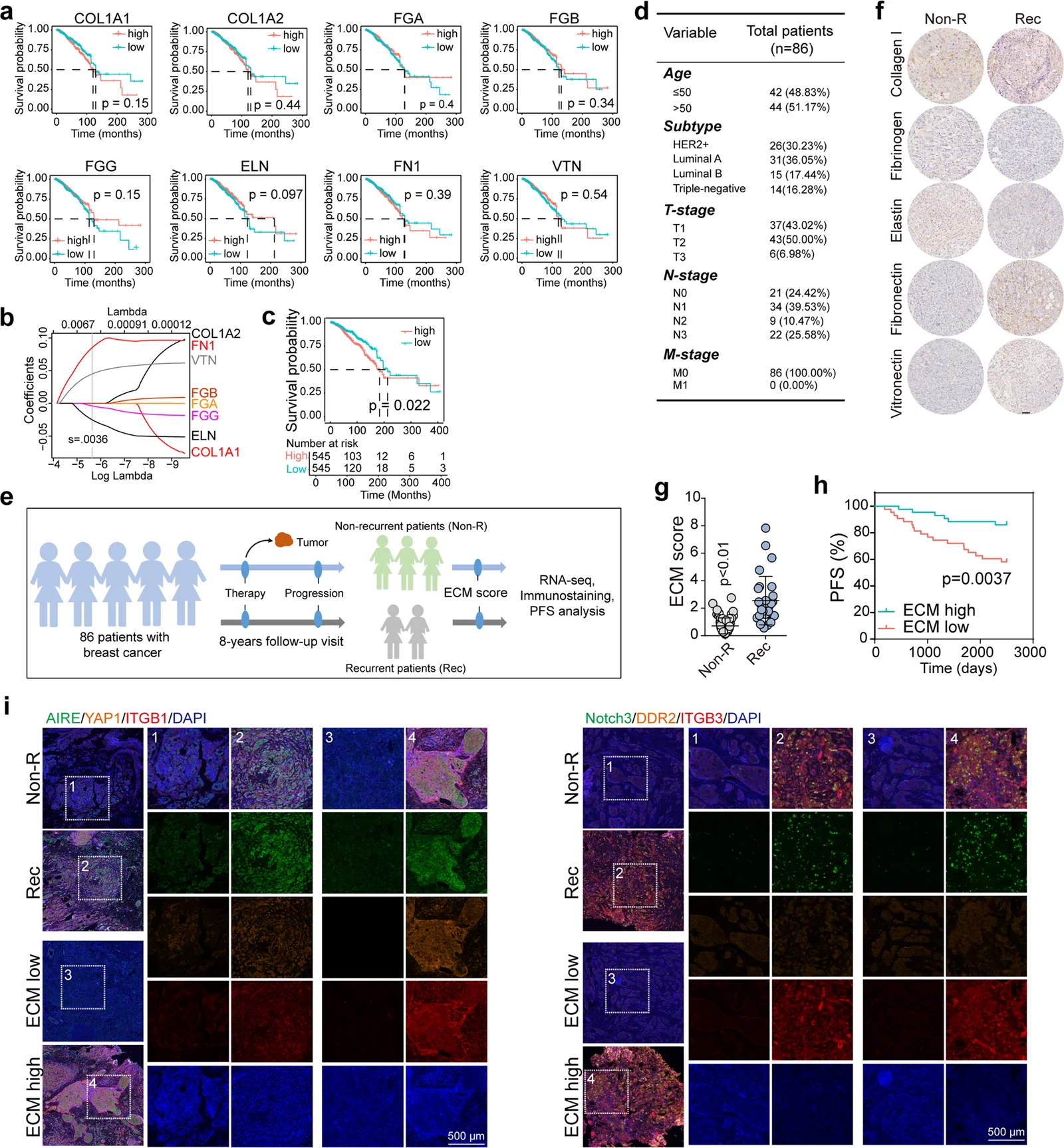
The novel ECM-score predicts clinical outcomes in patients with breast cancer. **a** Kaplan–Meier overall survival curves are shown according to the high and low expression of *COL1A1, COL1A2, FGA, FGB, FGG, ELN, FN1*, and *VTN* in 1358 patients with breast cancer, based on data obtained from TCGA. **b** LASSO coefficient profiles of eight genes (*COL1A1, COL1A2, FGA, FGB, FGG, ELN, FN1*, and *VTN*). **c** The Kaplan–Meier overall survival curve was shown according to the high and low ECM score in 1358 patients with breast cancer derived from TCGA data. **d** Information of 86 patients with breast cancer. **e** Tumor tissues were collected from the 86 patients after standard treatment. The patients were divided into recurrent and non-recurrent groups according to findings from an 8-year follow-up visit. **f** Immunohistochemistry of collagen I, fibrinogen, elastin, fibronectin, and vitronectin in tumor tissues from patients with recurrent and non-recurrent breast cancer. **g** The ECM score (protein level) was determined in 86 patients divided into the recurrent and non-recurrent groups. **h** The Kaplan–Meier overall survival curve was shown according to the high and low ECM scores (protein level) of 86 patients with breast cancer. **i** Immunostaining of AIRE, YAP1, ITGB1, ITGB3, Notch3 and DDR2 in tumor tissues divided into non-recurrent/recurrent or ECM high/low groups. The scale bar was 500 μm. Three independent experiments were performed. Data are represented as mean ± SEM. *P* < 0.05, statistical significance

Lastly, we investigated whether ECM composition could predict clinical outcomes in patients with breast cancer. Tumor cells could enter a dormant or quiescence-like state to escape apoptosis induced by chemotherapy or radiotherapy.^30,31^ Stem-like tumor cells serve as the critical driver during tumor recurrence and metastasis.^32^ Thus, we speculated that ECM induces a quiescent stem-like status in tumor cells and promotes their escape in clinical interventions via biomechanical force signals, eventually promoting tumor recurrence or distant metastasis after standard treatment. The ECM-induced biomechanical force is determined by the meshwork of fibers, including collagen, fibrinogen, elastin, fibronectin, and vitronectin. A total of 1358 patients with breast cancer were enrolled in our study. The expression data of ECM fiber-encoding genes (e.g., *COL1A1*, *COL1A2*, *FGA*, *FGB*, *FGG*, *ELN*, *FN1*, and *VTN*) from 1358 patients with breast cancer were used for Kaplan–Meier overall survival analysis. However, a single factor in the ECM cohort failed to influence the overall survival of patients with breast cancer (Fig. 6a).

Owing to the crosstalk between ECM compounds, we speculated that the biomechanical force might be determined by multitudinous ECM fibers and influenced by ECM composition, subsequently stimulating integrin and DDR signal activation in tumor cells. Thus, LASSO regression analysis was performed using ECM-encoding gene expression data to predict survival in the model (Fig. 6b). Finally, four genes were selected to construct the ECM signature. The ECM score was calculated using the following formula:

$${{{\mathrm{ECM}}}}\;{{{\mathrm{score}}}} = 0.0852 \times {{{\mathrm{Exp}}}}\left( {{{{\mathrm{FN}}}}1} \right) + 0.0449 \times {{{\mathrm{Exp}}}}\left( {{{{\mathrm{VTN}}}}} \right) - 0.0039 \times {{{\mathrm{Exp}}}}\left( {{{{\mathrm{FGG}}}}} \right) - 0.0252 \times {{{\mathrm{Exp}}}}\left( {{{{\mathrm{ELN}}}}} \right){{{\mathrm{.}}}}$$

Based on the ECM score, patients with breast cancer from the TCGA cohort were divided into high-score and low-score groups. The survival curve showed that patients with a low ECM score exhibited better overall survival than those with a high ECM score (Fig. 6c). To validate the prognostic value of our ECM score, 86 patients with breast cancer were enrolled (Fig. 6d) and divided into non-recurrent and recurrent groups according to the 8-year follow-up visit (Fig. 6e). The expression of collagen I, fibrinogen, elastin, fibronectin, and vitronectin in tumor tissues from patients with the recurrent and non-recurrent disease was determined using immunohistochemistry (Fig. 6f). The ECM score at the protein expression level in this validation set is shown in Fig. 6g, h. Consistent with this finding, patients with recurrent disease exhibited a considerable increase in the ECM score compared to patients with the non-recurrent disease (Fig. 6g). Meanwhile, patients in the high-score group showed a significantly poorer progression-free survival (PFS) than patients in the low-score group (Fig. 6h). Moreover, enhanced expression of stem-associated molecules, including integrin β1/3, Notch3, YAP1, and AIRE, was found in the recurrent and ECM-high groups compared to non-recurrent and ECM-low groups, respectively. Additionally, limited alterations of the cell quiescence-associated molecule DDR2 were observed (Fig. 6i). Collectively, these results suggest that ECM composition serves as a novel indicator to predict clinical outcomes in patients with breast cancer.

## Discussion

The ECM serves as a niche for cancer stem cells (CSCs) or tumor-initiating cells, which have the capability for self-renewal, tumor initiation, and drug resistance.^33,34^ The ECM provides biophysical and biochemical support to promote the proliferation, self-renewal, and differentiation of CSCs. This study revealed that ECM-derived biomechanical force was transduced by cytoskeleton prestress, which directly regulated cancer cell stemness and quiescence. Specifically, ECM-induced stress upregulated cancer stemness through integrin-cytoskeletal prestress and AIRE signals. In contrast, excessive stress promoted the transition to quiescent status in stem cell-like tumor cells through DDR/STAT1/P27 signaling. To our knowledge, our results are the first to reveal that ECM-induced mechano-transduction dynamically regulates cancer stemness and quiescence through dual signaling pathways. Our evidence highlights the crucial role of intracellular mechanical transduction and chromatin stretching in regulating cell function and fate.

Collagen I and laminin were previously reported to induce epithelial-mesenchymal transition, a major cellular transformation from a differentiated state to a stem cell-like state, by activating focal adhesion kinase signaling.^35–37^ Additionally, several ECM components, such as collagen, laminin, and fibronectin, reportedly preserve cancer cell stemness by activating transcriptional programs that induce self-renewal. For example, the binding of collagen to α2β1 integrin results in the activation of hedgehog signaling and the nuclear translocation of Bmi1, a stemness-inducing transcription factor.^38^ Laminin maintains breast cancer stemness through the activation of α6β1 integrin in a TAZ-dependent manner, leading to the upregulation of stemness transcription factors, such as OCT4, SOX2, and NANOG.^39^ Notably, the physical properties of ECM are crucial in regulating self-renewal and differentiation of CSCs. ECM stiffness directly promotes the ^40^ differentiation of human mesenchymal and neural stem cells into different cell lineages.^41,42^ However, controversial conclusions regarding matrix stiffness-mediated stemness in several cancers have also been reported. A soft external environment (3D fibrin gels) promotes stem gene expression and self-renewal in melanoma.^43^ In contrast, increased matrix stiffness increased CSC marker expression in breast cancer. Moreover, the mechanotransduction from ECM stiffness to intracellular prestresses and the underlying mechanism of ECM stiffness-regulated cancer stemness remain poorly understood.

Integrin-mediated signaling plays a crucial role in regulating cancer stemness and tumor development. Early studies have demonstrated that integrin receptors activate protein kinase B, a master regulator, via intracellular focal adhesion and integrin-linked kinases. This activates NF-κB and upregulates the expression of stemness genes *SOX2*, *NANOG*, and *KLF* in breast cancer and prostate cancer.^44^ Similarly, integrin αvβ3 reportedly activates Gli1 through a non-classic ERK1/2 pathway and the classic Hedgehog pathway to maintain CSC phenotypes in gastric cancer.^45^ Consistent with prior reports, we demonstrated that integrin β1 and β3 play key roles in collagen- and fibrinogen-regulated cancer stemness, respectively. Notably, we also revealed that integrin-cytoskeleton prestress directly activated AIRE, leading to the upregulation of multiple stem genes. We initially indicated the correlation between the integrin-cytoskeleton and AIRE signals and highlighted the role of AIRE in regulating breast tumor stemness. Notably, *AIRE* plays a vital role in immune tolerance and autoimmune diseases by regulating the expression of tissue-specific antigens in thymic epithelial cells.^46^ Recent studies also detected aberrant expression of AIRE in solid tumors, especially in breast cancers, and showed a close association between AIRE expression and breast cancer susceptibility and prognosis.^47,48^ Nevertheless, the underlying molecular mechanisms have not been investigated. This study demonstrates for the first time that AIRE plays a crucial role in ECM biomechanical force-regulated cancer stemness and tumor formation. AIRE is an essential DNA-binding protein with transcription-activating properties,^49^ and its mechanism of action of AIRE primarily relies on its physical availability and chromatin accessibility.^50^ Our results indicate that cytoskeleton prestress can physically activate AIRE and upregulate the expression of multiple stemness genes, especially *Wnt2*, *YAP1*, and *Notch*. However, the specific molecular mechanism of AIRE-induced stemness gene expression warrants further investigation. Evidence also indicates that AIRE interacts with the coactivator CREB-binding protein that regulates transcription factors, including NF-κΒ, STATs, and HIF-1α, which play vital roles in cancer stemness.^51^ Therefore, our findings highlight the crucial role of AIRE in cancer stemness and tumor formation, and points to a novel direction for future research.

The primary characteristic of CSCs is their ability to maintain a dormant or quiescent state, which is defined as reversible cell cycle arrest at the G0 phase in response to microenvironmental cues and therapeutic pressures. Generally, quiescence and stemness coexist in CSCs and normal stem cells. In hematopoietic stem cells, quiescence is crucial for facilitating long-term self-renewal.^52^ During tumor metastasis or relapse, disseminated cancer cells remain dormant or quiescent in distant organs for a long time and maintain their tumor initiation potential to form new metastatic colonies.^53^ However, the regulatory mechanisms underlying the transition from and to quiescent and active states in CSCs remain unclear. Our findings revealed that ECM-induced biomechanical force mediated the quiescence and activation of stem-like cells. Specifically, DDR2, a force signal receptor, was found to regulate the cell cycle arrest of breast cancer cells through the upregulation of STAT1/p27 signaling. This is consistent with the findings from a previous study that a collagen niche promoted cancer cell dormancy through the DDR/STAT axis in hepatocellular carcinoma. It explains why cancer cell dissemination exhibits a specific organ preference or organotropism, which is associated with different matrix stiffnesses in distinct organs. Notably, we observed that different strengths of force signals result in different outcomes for cell fate and function. Matrix stress of 30–90 Pa was found to upregulate cancer stemness. In contrast, excessive stress (>100 Pa) promoted the transition into quiescence in stem-like cells, which explicated controversial findings regarding the effect of matrix stiffness on tumor progression.

Our findings also suggest that ECM-induced biomechanical force plays a crucial role in cancer stemness and cell quiescence/palinesthesia. Cancer cells frequently enter a dormant or quiescent state to escape clinical intervention and apoptosis. These dormant cells can acquire stem-like phenotypes in the ECM microenvironment, leading to tumor recurrence and metastasis.^54^ Hence, we speculate that the ECM constitution, especially the fibrous components, is associated with tumor recurrence and prognosis in patients with breast cancer. In our study, we constructed and validated an ECM signature consisting of the expression of fibrinogen, elastin, fibronectin, and vitronectin to effectively predict clinical outcomes in patients with breast cancer. Notably, we demonstrated that a single component exhibited limited clinical significance in predicting patient survival. This suggests that the biomechanical force originating from the crosslinking of multifarious ECM components controls tumor development. This explains the conflicting results of the prognostic value of individual ECM components in previous studies. For instance, YK et al. found that fibronectin expression correlated with tumor aggressiveness and a poor clinical outcome in invasive breast cancers.^55^ In contrast, Shinde et al. reported that autocrine fibronectin expression inhibited breast cancer metastasis.^56^ Loa et al. demonstrated the poor relationship between collagen expression and overall survival in patients with breast cancer.^57^ A recent study also showed that collagen fiber orientation disorder was significantly associated with early-stage breast cancer prognosis.^58^ This validates the critical role of structure-associated biomechanical signals in tumor progression. Therefore, the ECM index developed in this study provides an additional advantage in predicting patient prognosis compared to using individual ECM components. We combined multiple ECM compounds, leading to a scientifically rigorous prediction method, and our findings were validated through mRNA-seq and protein expression analysis in patients with breast cancer. However, this study has some limitations. The specific mechanical force from each component should be thoroughly investigated, and the role of the ECM-induced biomechanical force may vary in tumors from different tissues. Despite these limitations, our findings provide novel and promising insights into predicting clinical outcomes in breast cancer.

In conclusion, our findings demonstrate that ECM-derived mechanical force governs breast cancer cell stemness and quiescence transition through independent integrin and DDR signaling. Additionally, our results suggest the presence of a novel ECM signature that can effectively predict the clinical prognosis of breast cancer.

## Materials and methods

### Clinical specimens

Eighty-six human breast tumor tissues were obtained from the Cancer Hospital, Chinese Academy of Medical Sciences, and categorized into non-recurrent (non-R, *n* = 61) and recurrent (Rec, *n* = 25) groups according to findings at the follow-up visit. All patients agreed to participate in the study and provided written informed consent. The experiments were performed in accordance with the guidelines of the Declaration of Helsinki. Ethical review was granted by the Ethics Committee of the Cancer Hospital, Chinese Academy of Medical Sciences. The transcriptome and survival information of 1358 patients with breast cancer was retrieved from the TCGA database.

### 3D gel culture

MCF-7 and 4T1 cells were seeded in 3D Matrigel, collagen I, and fibrinogen gels, as previously described.^17,43,59^ The substrate concentration was adjusted to produce 3D culture gels with different stiffnesses, as described previously.^43,60,61^ In our study, MCF-7 (5 × 10^4^ cells/well) or 4T1 (1 × 10^4^ cells/well) cells were seeded in a 24-well plate, with each well containing 1 mL of culture medium and 250 μL 3D gels. Cells were cultured in 3D gels for a maximum of 5 days. The 3D gels were degraded using dispase (Corning, USA) for cell isolation. For the tumorigenic assay, tumor cells were seeded in 3D Matrigel, collagen I, and fibrinogen gels (45 Pa). For the cell cycle assay, tumor cells were seeded in 3D Matrigel, collagen I, and fibrinogen gels (450 Pa). The morphology of the flask/3D cultured cells was analyzed using optical and atomic force microscopy (BRUKER, USA). The correlation between the gel stiffness and substance concentration is shown in the Supplementary information.

### Animal experiments

Female BalB/C and NOD-SCID mice (6 weeks old) were purchased from Vital River Laboratory Animal Technology Co. (China) and raised in a specific pathogen-free facility. Animal experiments were performed according to the guidelines of the Institute Ethics Committee of Cancer Hospital, Chinese Academy of Medical Sciences. To investigate the role of biomechanical force in governing cell quiescence/palinesthesia in vivo, 1 × 10^3^ 4T1 cells were encapsulated in 450 Pa 3D Matrigel (or not) and subcutaneously implanted in BalB/C mice (*n* = 10 in each group). On days 3 and 5, the mice were treated with PBS or dispase (for Matrigel degradation, 1 μL of dispase in 50 μL of PBS per mouse) via subcutaneous injection. On days 10 and 20, the mice were sacrificed, and skin samples from the site of tumor cell injection were collected for H&E staining. To generate the mouse model with DDR2-overexpressed MCF-7 cells, 1 × 10^6^ vector and DDR2-overexpressed MCF-7 cells were subcutaneously injected into NOD-SCID mice (*n* = 6 in each group). On day 15, tumor cells were isolated from tumor-bearing mice for cell cycle analysis. The tumor volume was recorded daily. Tumor volume was calculated using the following equation:

$${{{\mathrm{tumor}}}}\;{{{\mathrm{volume}}}} = {{{\mathrm{length}}}} \times {{{\mathrm{width}}}} \times {{{\mathrm{width}}}}^2/2.$$

### Statistical analysis

Data are presented as mean ± standard deviation (SD) and analyzed using GraphPad 5.0 (IBM, USA). The differences between the two groups were compared using an independent sample *t*-test. Comparisons among multiple groups were performed using one-way analysis of variance (ANOVA) followed by Tukey’s post-hoc test. The Kaplan–Meier estimator was used to evaluate the overall survival of the patients. Each experiment was performed with at least three independent rounds. Statistical significance was set at *p* < 0.05.

## Supplementary information

Sigtrans_Supplementary_Materials

## Data Availability

Data generated and analyzed in the present study are included in this manuscript and supplementary files. Additional information is available from the corresponding author on reasonable request.

## References

[1] Mohan, V., Das, A. & Sagi, I. Emerging roles of ECM remodeling processes in cancer. *Semin Cancer Biol* **62**, 192–200 (2020). 10.1016/j.semcancer.2019.09.004 31518697

[2] Fang, M., Yuan, J., Peng, C. & Li, Y. Collagen as a double-edged sword in tumor progression. *Tumour Biol* **35**, 2871–2882 (2014). 10.1007/s13277-013-1511-7 PMC3980040 24338768

[3] Girigoswami, K., Saini, D. & Girigoswami, A. Extracellular matrix remodeling and development of cancer. *Stem Cell Rev Rep* **17**, 739–747 (2021). 10.1007/s12015-020-10070-1 33128168

[4] Li, C. L. et al. Fibronectin induces epithelial-mesenchymal transition in human breast cancer MCF-7 cells via activation of calpain. *Oncol Lett* **13**, 3889–3895 (2017). 10.3892/ol.2017.5896 PMC5431265 28521486

[5] Shields, M. A., Dangi-Garimella, S., Redig, A. J. & Munshi, H. G. Biochemical role of the collagen-rich tumour microenvironment in pancreatic cancer progression. *Biochem J* **441**, 541–552 (2012). 10.1042/BJ20111240 PMC8215985 22187935

[6] Iozzo, R. V. & Sanderson, R. D. Proteoglycans in cancer biology, tumour microenvironment and angiogenesis. *J Cell Mol Med* **15**, 1013–1031 (2011). 10.1111/j.1582-4934.2010.01236.x PMC3633488 21155971

[7] Liu, X. et al. Metastatic breast cancer cells overexpress and secrete miR-218 to regulate type I collagen deposition by osteoblasts. *Breast Cancer Res* **20**, 127 (2018). 10.1186/s13058-018-1059-y PMC6198446 30348200

[8] Xu, S. et al. The role of collagen in cancer: from bench to bedside. *J Transl Med* **17**, 309 (2019). 10.1186/s12967-019-2058-1 PMC6744664 31521169

[9] Nallanthighal, S., Heiserman, J. P. & Cheon, D. J. The role of the extracellular matrix in cancer stemness. *Front Cell Dev Biol* **7**, 86 (2019). 10.3389/fcell.2019.00086 PMC6624409 31334229

[10] Jokela, T. A. et al. Microenvironment-induced non-sporadic expression of the AXL and cKIT receptors are related to epithelial plasticity and drug resistance. *Front Cell Dev Biol* **6**, 41 (2018). 10.3389/fcell.2018.00041 PMC5913284 29719832

[11] Yu, Q. et al. Fibronectin promotes the malignancy of glioma stem-like cells via modulation of cell adhesion, differentiation, proliferation and chemoresistance. *Front Mol Neurosci* **11**, 130 (2018). 10.3389/fnmol.2018.00130 PMC5908975 29706869

[12] Vaidyanath, A. et al. Hyaluronic acid mediated enrichment of CD44 expressing glioblastoma stem cells in U251MG xenograft mouse model. *J Stem Cell Res Ther* **7**, 2 (2017).

[13] Gattazzo, F., Urciuolo, A. & Bonaldo, P. Extracellular matrix: a dynamic microenvironment for stem cell niche. *Biochim Biophys Acta Gen Subj* **1840**, 2506–2519 (2014). 10.1016/j.bbagen.2014.01.010 PMC4081568 24418517

[14] Aguirre-Ghiso, J. A., Estrada, Y., Liu, D. & Ossowski, L. ERK(MAPK) activity as a determinant of tumor growth and dormancy; regulation by p38(SAPK). *Cancer Res* **63**, 1684–1695 (2003). 12670923

[15] Barney, L. E. et al. Tumor cell-organized fibronectin maintenance of a dormant breast cancer population. *Sci Adv* **6**, eaaz4157 (2020). 10.1126/sciadv.aaz4157 PMC7065904 32195352

[16] Barkan, D. et al. Metastatic growth from dormant cells induced by a col-I-enriched fibrotic environment. *Cancer Res* **70**, 5706–5716 (2010). 10.1158/0008-5472.CAN-09-2356 PMC3436125 20570886

[17] Qiu, Y. et al. Biomaterial 3D collagen I gel culture model: a novel approach to investigate tumorigenesis and dormancy of bladder cancer cells induced by tumor microenvironment. *Biomaterials* **256**, 120217 (2020). 10.1016/j.biomaterials.2020.120217 32736172

[18] Hadden, M. et al. Mechanically stressed cancer microenvironment: Role in pancreatic cancer progression. *Biochim Biophys Acta Rev Cancer* **1874**, 188418 (2020). 10.1016/j.bbcan.2020.188418 32827581

[19] Liu, C., Pei, H. & Tan, F. Matrix stiffness and colorectal cancer. *Onco Targets Ther* **13**, 2747–2755 (2020). 10.2147/OTT.S231010 PMC7131993 32280247

[20] Liu, Y. et al. Fibrin stiffness mediates dormancy of tumor-repopulating cells via a Cdc42-driven Tet2 epigenetic programMatrix stiffness induces TRC dormancy. *Cancer Res* **78**, 3926–3937 (2018). 10.1158/0008-5472.CAN-17-3719 29764867

[21] Gan, L. et al. Extracellular matrix protein 1 promotes cell metastasis and glucose metabolism by inducing integrin β4/FAK/SOX2/HIF-1α signaling pathway in gastric cancer. *Oncogene* **37**, 744–755 (2018). 10.1038/onc.2017.363 29059156

[22] Li, W., Liu, Z., Zhao, C. & Zhai, L. Binding of MMP-9-degraded fibronectin to β6 integrin promotes invasion via the FAK-Src-related Erk1/2 and PI3K/Akt/Smad-1/5/8 pathways in breast cancer. *Oncol Rep* **34**, 1345–1352 (2015). 10.3892/or.2015.4103 26134759

[23] Panciera, T. et al. Reprogramming normal cells into tumour precursors requires ECM stiffness and oncogene-mediated changes of cell mechanical properties. *Nat Mater* **19**, 797–806 (2020). 10.1038/s41563-020-0615-x PMC7316573 32066931

[24] Chowdhury, F., Huang, B. & Wang, N. Cytoskeletal prestress: the cellular hallmark in mechanobiology and mechanomedicine. *Cytoskeleton (Hoboken)* **78**, 249–276 (2021). 10.1002/cm.21658 PMC8518377 33754478

[25] Kechagia, J. Z., Ivaska, J. & Roca-Cusachs, P. Integrins as biomechanical sensors of the microenvironment. *Nat Rev Mol Cell Biol* **20**, 457–473 (2019). 10.1038/s41580-019-0134-2 31182865

[26] Barczyk, M., Carracedo, S. & Gullberg, D. Integrins. *Cell Tissue Res* **339**, 269–280 (2010). 10.1007/s00441-009-0834-6 PMC2784866 19693543

[27] Liu, Y. et al. Autoimmune regulator expression in thymomas with or without autoimmune disease. *Immunol Lett* **161**, 50–56 (2014). 10.1016/j.imlet.2014.04.008 24768600

[28] Abramson, J. & Husebye, E. S. Autoimmune regulator and self-tolerance—molecular and clinical aspects. *Immunol Rev* **271**, 127–140 (2016). 10.1111/imr.12419 27088911

[29] Di Martino, J. S. et al. A tumor-derived type III collagen-rich ECM niche regulates tumor cell dormancy. *Nat Cancer* **3**, 90–107 (2022). 10.1038/s43018-021-00291-9 PMC8818089 35121989

[30] De Angelis, M. L., Francescangeli, F., La Torre, F. & Zeuner, A. Stem cell plasticity and dormancy in the development of cancer therapy resistance. *Front Oncol* **9**, 626 (2019). 10.3389/fonc.2019.00626 PMC6636659 31355143

[31] Ebinger, S. et al. Characterization of rare, dormant, and therapy-resistant cells in acute lymphoblastic Leukemia. *Cancer Cell* **30**, 849–862 (2016). 10.1016/j.ccell.2016.11.002 PMC5156313 27916615

[32] Peitzsch, C., Tyutyunnykova, A., Pantel, K. & Dubrovska, A. Cancer stem cells: the root of tumor recurrence and metastases. *Semin Cancer Biol* **44**, 10–24 (2017). 10.1016/j.semcancer.2017.02.011 28257956

[33] Brown, Y., Hua, S. & Tanwar, P. S. Extracellular matrix-mediated regulation of cancer stem cells and chemoresistance. *Int J Biochem Cell Biol* **109**, 90–104 (2019). 10.1016/j.biocel.2019.02.002 30743057

[34] Reinhard, J., Brösicke, N., Theocharidis, U. & Faissner, A. The extracellular matrix niche microenvironment of neural and cancer stem cells in the brain. *Int J Biochem Cell Biol* **81**, 174–183 (2016). 10.1016/j.biocel.2016.05.002 27157088

[35] Shintani, Y., Maeda, M., Chaika, N., Johnson, K. R. & Wheelock, M. J. Collagen I promotes epithelial-to-mesenchymal transition in lung cancer cells via transforming growth factor-beta signaling. *Am J Respir Cell Mol Biol* **38**, 95–104 (2008). 10.1165/rcmb.2007-0071OC PMC2176131 17673689

[36] Begum, A. et al. The extracellular matrix and focal adhesion kinase signaling regulate cancer stem cell function in pancreatic ductal adenocarcinoma. *PLoS One* **12**, e0180181 (2017). 10.1371/journal.pone.0180181 PMC5503247 28692661

[37] Govaere, O. et al. Laminin-332 sustains chemoresistance and quiescence as part of the human hepatic cancer stem cell niche. *J Hepatol* **64**, 609–617 (2016). 10.1016/j.jhep.2015.11.011 26592953

[38] Suh, H. N. & Han, H. J. Collagen I regulates the self-renewal of mouse embryonic stem cells through α2β1 integrin- and DDR1-dependent Bmi-1. *J Cell Physiol* **226**, 3422–3432 (2011). 10.1002/jcp.22697 21344393

[39] Chang, C. et al. A laminin 511 matrix is regulated by TAZ and functions as the ligand for the α6Bβ1 integrin to sustain breast cancer stem cells. *Genes Dev* **29**, 1–6 (2015). 10.1101/gad.253682.114 PMC4281560 25561492

[40] Shah, L., Latif, A., Williams, K. J. & Tirella, A. Role of stiffness and physico-chemical properties of tumour microenvironment on breast cancer cell stemness. *Acta Biomater* **152**, 273–289 (2022). 10.1016/j.actbio.2022.08.074 36087866

[41] Islam, A., Mbimba, T., Younesi, M. & Akkus, O. Effects of substrate stiffness on the tenoinduction of human mesenchymal stem cells. *Acta Biomater* **58**, 244–253 (2017). 10.1016/j.actbio.2017.05.058 PMC5551443 28602855

[42] Rammensee, S., Kang, M. S., Georgiou, K., Kumar, S. & Schaffer, D. V. Dynamics of mechanosensitive neural stem cell differentiation. *Stem Cells* **35**, 497–506 (2017). 10.1002/stem.2489 PMC5285406 27573749

[43] Liu, J. et al. Soft fibrin gels promote selection and growth of tumorigenic cells. *Nat Mater* **11**, 734–741 (2012). 10.1038/nmat3361 PMC3405191 22751180

[44] Xiong, J. et al. Integrins regulate stemness in solid tumor: an emerging therapeutic target. *J Hematol Oncol* **14**, 177 (2021). 10.1186/s13045-021-01192-1 PMC8555177 34715893

[45] Dong, H. et al. GLI1 activation by non-classical pathway integrin α(v)β(3)/ERK1/2 maintains stem cell-like phenotype of multicellular aggregates in gastric cancer peritoneal metastasis. *Cell Death Dis* **10**, 574 (2019). 10.1038/s41419-019-1776-x PMC6668446 31366904

[46] Yano, M. et al. Aire controls the differentiation program of thymic epithelial cells in the medulla for the establishment of self-tolerance. *J Exp Med* **205**, 2827–2838 (2008). 10.1084/jem.20080046 PMC2585853 19015306

[47] Bianchi, F. et al. Expression and prognostic significance of the autoimmune regulator gene in breast cancer cells. *Cell Cycle* **15**, 3220–3229 (2016). 10.1080/15384101.2016.1241918 PMC5176139 27753538

[48] Fawzy, M. S. & Toraih, E. A. Analysis of the autoimmune regulator (AIRE) gene variant rs2075876 (G/A) association with breast cancer susceptibility. *J Clin Lab Anal* **34**, e23365 (2020). 10.1002/jcla.23365 PMC7521301 32426878

[49] Kumar, P. G. et al. The autoimmune regulator (AIRE) is a DNA-binding protein. *J Biol Chem* **276**, 41357–41364 (2001). 10.1074/jbc.M104898200 11533054

[50] Perniola, R. & Musco, G. The biophysical and biochemical properties of the autoimmune regulator (AIRE) protein. *Biochim Biophys Acta* **1842**, 326–337 (2014). 10.1016/j.bbadis.2013.11.020 24275490

[51] Pitkänen, J. et al. The autoimmune regulator protein has transcriptional transactivating properties and interacts with the common coactivator CREB-binding protein. *J Biol Chem* **275**, 16802–16809 (2000). 10.1074/jbc.M908944199 10748110

[52] Shin, J. J. et al. Controlled cycling and quiescence enables efficient HDR in engraftment-enriched adult hematopoietic stem and progenitor cells. *Cell Rep* **32**, 108093 (2020). 10.1016/j.celrep.2020.108093 PMC7487781 32877675

[53] Chen, W., Dong, J., Haiech, J., Kilhoffer, M. C. & Zeniou, M. Cancer stem cell quiescence and plasticity as major challenges in cancer therapy. *Stem Cells Int* **2016**, 1740936 (2016). 10.1155/2016/1740936 PMC4932171 27418931

[54] Sistigu, A., Musella, M., Galassi, C., Vitale, I. & De Maria, R. Tuning cancer fate: tumor microenvironment’s role in cancer stem cell quiescence and reawakening. *Front Immunol* **11**, 2166 (2020). 10.3389/fimmu.2020.02166 PMC7609361 33193295

[55] Bae, Y. K. et al. Fibronectin expression in carcinoma cells correlates with tumor aggressiveness and poor clinical outcome in patients with invasive breast cancer. *Hum Pathol* **44**, 2028–2037 (2013). 10.1016/j.humpath.2013.03.006 23684510

[56] Shinde, A. et al. Autocrine fibronectin inhibits breast cancer metastasis. *Mol Cancer Res* **16**, 1579–1589 (2018). 10.1158/1541-7786.MCR-18-0151 PMC6511995 29934326

[57] Ioachim, E. et al. Immunohistochemical expression of extracellular matrix components tenascin, fibronectin, collagen type IV and laminin in breast cancer: their prognostic value and role in tumour invasion and progression. *Eur J Cancer* **38**, 2362–2370 (2002). 10.1016/s0959-8049(02)00210-1 12460779

[58] Li, H. et al. Collagen fiber orientation disorder from H&E images is prognostic for early stage breast cancer: clinical trial validation. *NPJ Breast Cancer* **7**, 104 (2021). 10.1038/s41523-021-00310-z PMC8346522 34362928

[59] Yan, W. et al. A three-dimensional culture system with matrigel promotes purified spiral ganglion neuron survival and function in vitro. *Mol Neurobiol* **55**, 2070–2084 (2018). 10.1007/s12035-017-0471-0 28283883

[60] Riedl, P., Schricker, M. & Pompe, T. Stiffness variation of 3D collagen networks by surface functionalization of network Fibrils with sulfonated polymers. *Gels* **7**, 266 (2021). 10.3390/gels7040266 PMC8702206 34940326

[61] Soofi, S. S., Last, J. A., Liliensiek, S. J., Nealey, P. F. & Murphy, C. J. The elastic modulus of Matrigel as determined by atomic force microscopy. *J Struct Biol* **167**, 216–219 (2009). 10.1016/j.jsb.2009.05.005 PMC2747304 19481153

