# Correction: Extracellular matrix-derived mechanical force governs breast cancer cell stemness and quiescence transition through integrin-DDR signaling

**DOI:** 10.1038/s41392-026-03031-6

**Published:** 2026-09-24

**Authors:** Cong Li, Shi Qiu, Xiaohan Liu, Fengzhu Guo, Jingtong Zhai, Zhijun Li, Linghui Deng, Liming Ge, Haili Qian, Lu Yang, Binghe Xu

**Affiliations:** 1 Department of Medical Oncology, National Cancer Center/National Clinical Research Center for Cancer/Cancer Hospital, Chinese Academy of Medical Sciences and Peking Union Medical College, Beijing, 100021 China; 2 State Key Laboratory of Molecular Oncology, National Cancer Center/National Clinical Research Center for Cancer/Cancer Hospital, Chinese Academy of Medical Sciences and Peking Union Medical College, Beijing, 100021 China; 3 Department of Urology, Institute of Urology and National Clinical Research Center for Geriatrics, West China Hospital of Sichuan University, Chengdu, 610041 China; 4 National Clinical Research Center of Geriatrics, The Center of Gerontology and Geriatrics, West China Hospital, Sichuan University, Chengdu, 610065 China; 5 Institute of Oncology Research (IOR), Oncology Institute of Southern Switzerland (IOSI), Bellinzona, 6500 Switzerland; 6 Department of Histology and Embryology, Basic Medical College, China Medical University, Shenyang, Liaoning 110122 China; 7 Department of Pharmaceutical and Bioengineering, School of Chemical Engineering, Sichuan University, Chengdu, 610065 China

Correction to: *Signal Transduction and Targeted Therapy* https://doi.org/10.1038/s41392-023-01453-0, published online 28 June 2023

After online publication of the article,^1^ it was noticed that the immunohistochemistry images of the Flask group at Day 10 and Day 20 are partially overlapped in Figure 4f. During the figure assembly process, an image from a different field of view for Day 10 was mistakenly used for the Day 20 panel, resulting in unintended duplication between the two panels. The authors have re-examined the original raw data and located the correct Day 20 image, which has now been properly replaced. This correction does not affect any of the quantitative analyses, statistical outcomes, or scientific conclusions of our study. The correct Figure 4f were provided as follows.

Incorrect figure 4

**Fig. 4:**
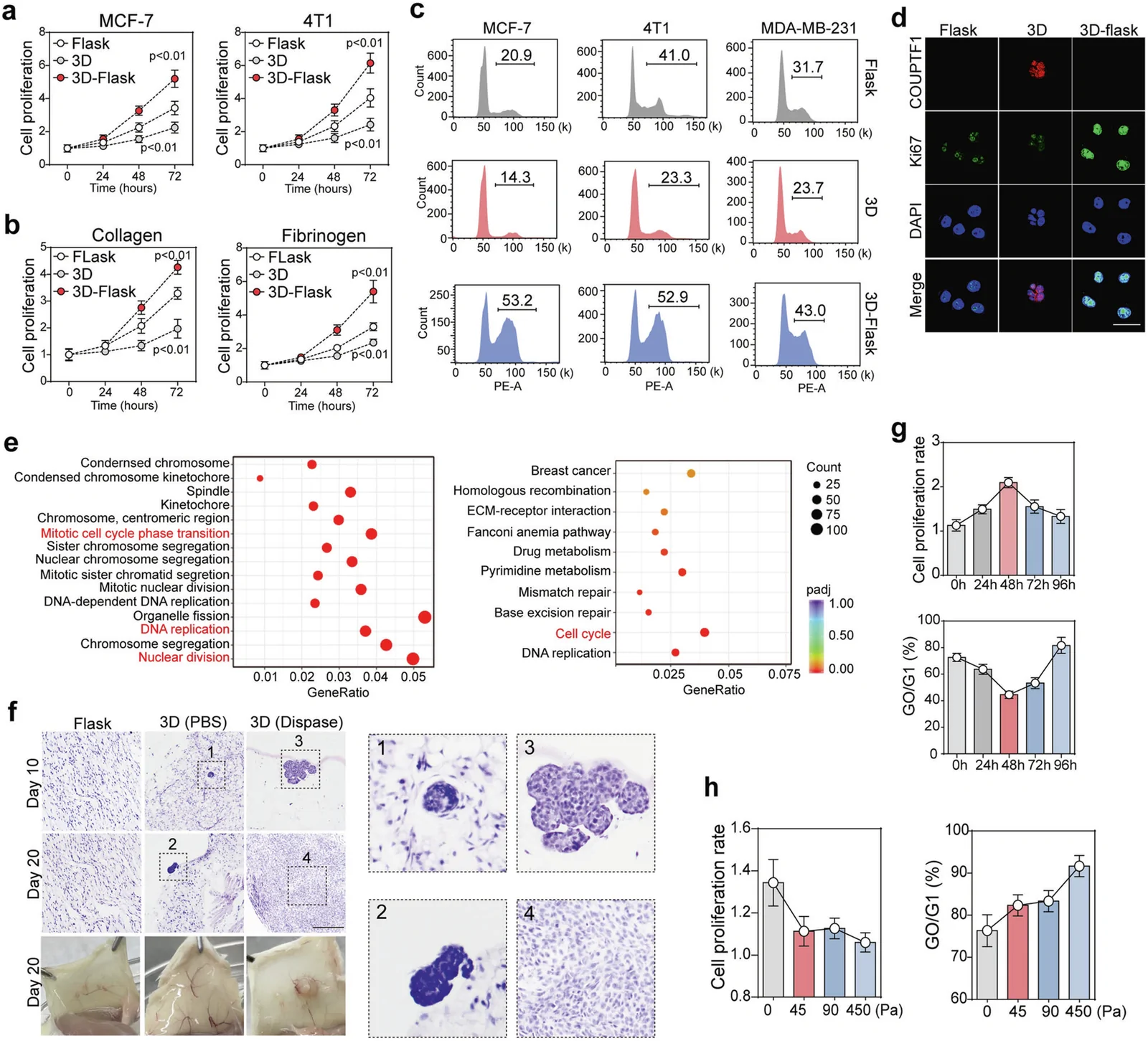
ECM-induced biomechanical force drives stem cell-like tumor cell quiescence. **a** MCF-7/4T1 cells were seeded in a flask and 3D Matrigel, following which the cells were harvested for the cell proliferation assay in a 96-well plate (flask and 3D-flask groups). Some of the 3D-cultured MCF-7/4T1 cells were re-seeded in 3D Matrigel and subjected to cell proliferation determination at the same time points (3D group). **b** Proliferation of MCF-7 cells cultured in a flask, 3D-flask, and 3D gel (3D collagen and fibrinogen culture system). **c** Cell cycle analysis of MCF7/4T1/MDA-MB-231 cells cultured in a flask, 3D gels, and 3D-flask. **d** Immunostaining for Ki67 and CoupTF1 in the flask, 3D gel, and 3D-flask groups. The scale bar is 20 μm. **e** GO and KEGG enrichment analysis of differentially expressed genes in the 4T1 cells cultured in the flask and 3D culture system, with a significance threshold of p-value < 0.05. **f** 1 × 10^3^ 4T1 cells were encapsulated in a 450-Pa 3D Matrigel (or not) and subcutaneously implanted into mice. On days 3 and 5, the mice were treated with PBS or dispase administered via subcutaneous injection. H&E staining of the hypodermis in each group was performed on days 10 and 20 (*n* = 10). The scale bar was 500 μm. **g** MCF-7 cells were cultured in the 3D Matrigel for 3 days and isolated for flask culture. After 0, 24, 48, 72, and 96 h of flask culture, cell proliferation or cycle was examined. **h** MCF-7 cells were seeded in 45-, 90-, and 450-Pa Matrigel for 3 days. Cell cycle and proliferation (in Matrigel) were examined. Three independent experiments were performed. Data are represented as mean ± SEM. *P* < 0.05, statistical significance

correct figure 4

**Fig. 4:**
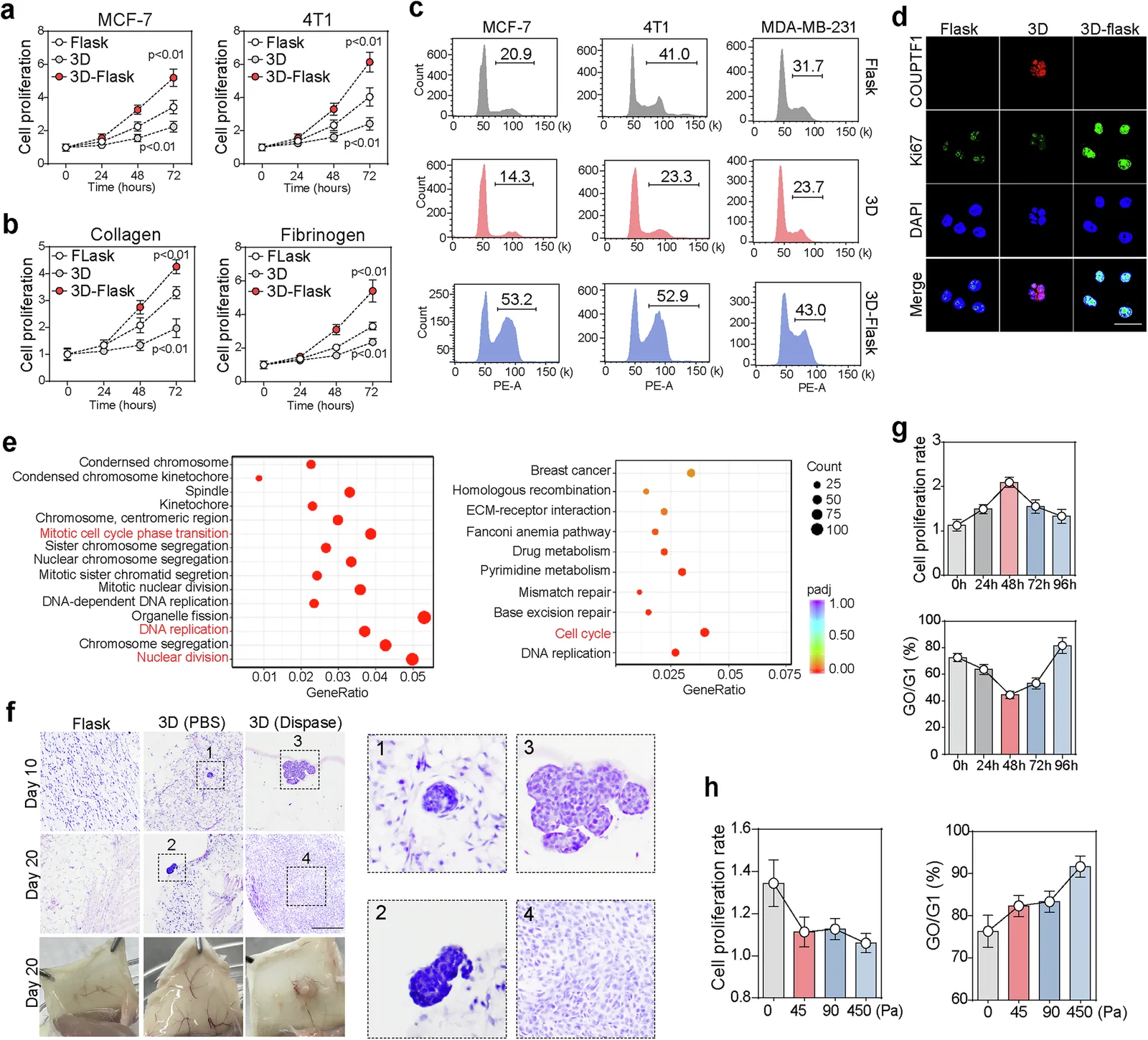
ECM-induced biomechanical force drives stem cell-like tumor cell quiescence. **a** MCF-7/4T1 cells were seeded in a flask and 3D Matrigel, following which the cells were harvested for the cell proliferation assay in a 96-well plate (flask and 3D-flask groups). Some of the 3D-cultured MCF-7/4T1 cells were re-seeded in 3D Matrigel and subjected to cell proliferation determination at the same time points (3D group). **b** Proliferation of MCF-7 cells cultured in a flask, 3D-flask, and 3D gel (3D collagen and fibrinogen culture system). **c** Cell cycle analysis of MCF7/4T1/MDA-MB-231 cells cultured in a flask, 3D gels, and 3D-flask. **d** Immunostaining for Ki67 and CoupTF1 in the flask, 3D gel, and 3D-flask groups. The scale bar is 20 μm. **e** GO and KEGG enrichment analysis of differentially expressed genes in the 4T1 cells cultured in the flask and 3D culture system, with a significance threshold of p-value < 0.05. **f** 1 × 10^3^ 4T1 cells were encapsulated in a 450-Pa 3D Matrigel (or not) and subcutaneously implanted into mice. On days 3 and 5, the mice were treated with PBS or dispase administered via subcutaneous injection. H&E staining of the hypodermis in each group was performed on days 10 and 20 (*n* = 10). The scale bar was 500 μm. **g** MCF-7 cells were cultured in the 3D Matrigel for 3 days and isolated for flask culture. After 0, 24, 48, 72, and 96 h of flask culture, cell proliferation or cycle was examined. **h** MCF-7 cells were seeded in 45-, 90-, and 450-Pa Matrigel for 3 days. Cell cycle and proliferation (in Matrigel) were examined. Three independent experiments were performed. Data are represented as mean ± SEM. *P* < 0.05, statistical significance
