# Supplementary material for: Extracellular matrix-derived mechanical force governs breast cancer cell stemness and quiescence transition through integrin-DDR signaling
Source: Signal Transduct Target Ther. 2023 Jun 28;8:247. doi: 10.1038/s41392-023-01453-0 (PMC10300038; doi:10.1038/s41392-023-01453-0)
Supplement: Supplementary file 1 — Sigtrans_Supplementary_Materials [file 41392_2023_1453_MOESM1_ESM.docx]

Supplementary Materials for

Extracellular matrix-derived mechanical force governs breast cancer cell stemness and quiescence transition through integrin-DDR signaling

Cong Li^1,2*^, Shi Qiu^3,7*^, Xiaohan Liu^6*^, Fengzhu Guo^1,2^, Jingtong Zhai^1^, Zhijun Li^1^, Linghui Deng^7^, Liming Ge^5^, Haili Qian^2^**^#^**, Lu Yang^3^**^#^**, Binghe Xu^1^**^#^**

Correspondence to: Binghe Xu (xubingheBM@163.com), Haili Qian (qianhaili001@163.com), and Lu Yang (wycleflue@163.com)

**This PDF file includes:**

Materials and Methods

Figures. S1 to S5

Materials and Methods

Cell culture and reagents

Human breast cancer cell line MCF-7, human breast cancer cell line MDA-MB-231 and murine breast cancer cell line 4T1 were obtained from American Type Culture Collection (USA) and maintained in Roswell Park Memorial Institute 1640 culture medium containing 10% fetal bovine serum in a 5% CO_2_ incubator at 37℃. DDR2 or AIRE overexpressed MCF-7, 4T1 and MDA-MB-231 cell lines were obtained from Cyagen (China). pCMV-AIRE-3×FLAG and pCMV-DDR2-3×FLAG vectors were transfected using Lipofect8000 (Thermo Fisher, USA). Cytoskeleton inhibitor 5a-Pregnane-3,20-dione was obtained from Selleck Co. (USA). Integrin β1 and β3 neutralizing antibodies were obtained from Solarbio Co. (China). Type I collagen and fibrinogen gels were obtained from Solarbio (China). Matrigel and Dispase were obtained from Corning (USA). YAP1 inhibitor YAP-TEAD-IN-1 and Notch inhibitor 1 were obtained from MedChemExpress Co. (USA).

3D gel concentration

The gel stiffness (biomechanical force) was determined by the substance concentration:

| Gel stiffness (Pa) | Collagen (mg/ml) | Fibrin (mg/ml) | Matrigel (protein concentration, mg/ml) |
| --- | --- | --- | --- |
| 15 | 0.3 | 0.12 | 3.9 |
| 30 | 0.4 | 0.21 | 4.3 |
| 45 | 0.9 | 0.37 | 6.5 |
| 90 | 3.1 | 0.9 | 11.3 |
| 450 | 6.7 | 4.3 | 14.2 |
| 1050 | 7.3 | 10.2 | 17.2 |

Cell proliferation

Cell proliferation was determined by cell counting kit-8 (CCK8, Biyuntian, China). Briefly, flask or 3D cultured tumor cells were isolated and seeded into a 96-well plate (3×10^3^ cells/well). After 0, 24, 48 and 72 hours, the cells were incubated with 10% CCK-8 solution for 3 hours. The absorbance at 450 nm was detected using a microplate reader (Thermo Fisher, USA).

Tumorigenic potential assay

Tumorigenic potential was determined by *in vitro* colony formation and *in vivo* subcutaneous neoplasia assay. For *in vitro* colony formation, 200 MCF-7, 4T1 or MDA-MB-231 cells were seeded in agarose soft gel in 24-well plate. After 14 days, colonies were fixed with paraformaldehyde and stained with crystal violet. Colonies (>50 cells) were counted under optical microscopy (BRUKER, USA). For *in vivo* subcutaneous neoplasia, 1×10^5^ flask cultured MCF-7 or 1×10^4^ flask cultured 4T1/MDA-MB-231 cells were digested, resuspended in PBS and subcutaneously injected into the immunodeficient mice. For 3D cultured cells, 1×10^5^ 3D cultured MCF-7 or 1×10^4^ 3D cultured 4T1/MDA-MB-231 cells were seeded into the 3D gel in a 96-well plate (50 μl) again. After 2 hours, the solid gels containing cells were implanted under the shin of immunodeficient mice (n=10 in each group). Tumor formation was counted on day 20. Each experiment was repeated for three independent time.

Cell cycle assay

Cell cycle of tumor cells was determined by propidium iodide (PI) staining. Briefly, 3D or flask cultured tumor cells were washed and fixed with cold 70% ethanol for at least one hour. Then cells were stained with propidium iodide staining solution containing RNase A stock solution (Becton, Dickinson and Company, USA) overnight at 4 ℃. Cell cycle was analyzed by flow cytometry (Becton, Dickinson and Company, USA).

Flow cytometry.

Tumor cells were digested to single cells using 0.05% trypsin and then incubated with FITC-ALDH1analysis kit (BD Biosciences, USA). Analysis was performed using a C6 flow cytometer (BD Biosciences, USA).

RNA interference

SiRNA targeting AIRE was obtained from Tsingke (China) (siRNA #1: 5′-GGACCAAUCUCCGCUGCAATT -3′ and siRNA#2: 5’-GAGUCAGGAAGAUCCAAGATT -3’) Transfection of siRNA into cells was performed using Lipofectamine® 8000 reagent (Thermo Fisher, USA) at a final concentration of 20 nM. The silence efficiency was examined by qPCR.

Real-time quantitative polymerase chain reaction (qPCR)

Total RNA extracted from tumor cells was reversely transcribed into cDNA with a reverse transcription kit (Thermo Fisher USA). Quantified PCR was performed by applying the SYBR Green Real-Time PCR Master Mixes (Thermo Fisher, USA) on an ABI7200 instrument (Applied Biosystems, USA). The primer sequences were downloaded from https://pga.mgh.harvard.edu/primerbank/ and synthesized by Sangon (China). Glyceraldehyde3-phosphate dehydrogenase (GAPDH) was used as internal control.

Western blotting

Total proteins were extracted from MCF-7, 4T1 or MDA-MB-231 cells using lysis buffer (Thermo Fisher, USA). Proteins were separated by PAGE with 10% separating gel and transferred onto a nitrocellulose membrane (Beyotime Biotechnology, China) using wet transfer apparatus. Samples were blocked with bovine serum albumin for 30 minutes at room temperature and incubated with primary antibodies overnight at 4°C: anti-integrin β1 (ab179471, Abcam, UK), anti-integrin β3 (ab179473, Abcam, UK), anti-GAPDH (ab8245, Abcam, UK), anti-AIRE (ab65040, Abcam, UK), anti-DDR1 (#3917s, Cell signaling Technology, USA), anti-DDR2 (#12133s, Cell signaling Technology, USA), anti-STAT1 (ab239360, Abcam, UK), anti-phosphorylated STAT1 (ab109461, Abcam, UK), anti-P27 (ab32034, Abcam, UK), anti-P57 (ab119989, Abcam, UK), anti-P21 (ab109520, Abcam, UK), anti-YAP1 (ab52771, Abcam, UK), anti-Histone H3 (ab1791, Abcam, UK)), anti-Notch3 (ab23426, Abcam, UK) and anti-Notch4 (ab184742, Abcam, UK). The membranes were further incubated by secondary antibodies for 2 hours and visualized in a gel imaging system (Thermo Fisher, USA).

Immunostaining

Sections of breast tumor tissues were treated with 2.5% H_2_O_2_ and 5% bovine serum albumin for 30 minutes at room temperature. Samples were incubated with primary antibodies: anti-collagen I (ab138492, Abcam, UK), anti-fibrinogen (ab281924, Abcam, UK), anti-fibronectin (ab2413, Abcam, UK), anti-elastin (ab9519, Abcam, UK), anti-vitronectin (ab46808, Abcam, UK), anti-Notch3 (ab23426, Abcam, UK), anti-YAP1 (ab52771, Abcam, UK), anti-AIRE (ab65040, Abcam, UK), anti-integrin β1 (ab179471, Abcam, UK), anti-integrin β3 (ab179473, Abcam, UK) and anti-DDR2 (#12133s, Cell signaling Technology, USA) overnight at 4°C. MCF-7, 4T1 and MDA-MB-231 cells were incubated with anti-F-actin primary antibody (ab205, Abcam, UK) for cytoskeleton study. Following incubation with horseradish peroxidase-conjugated secondary antibodies for one hour at room temperature, sections were then visualized and analyzed under optical microscope (Leica, Germany) or confocal microscope (Olympus, Germany). Protein expression was quantified by Image-Pro Plus 5.0 software (MEDIA CYBERNETICS, USA).

Figure. S1.

**
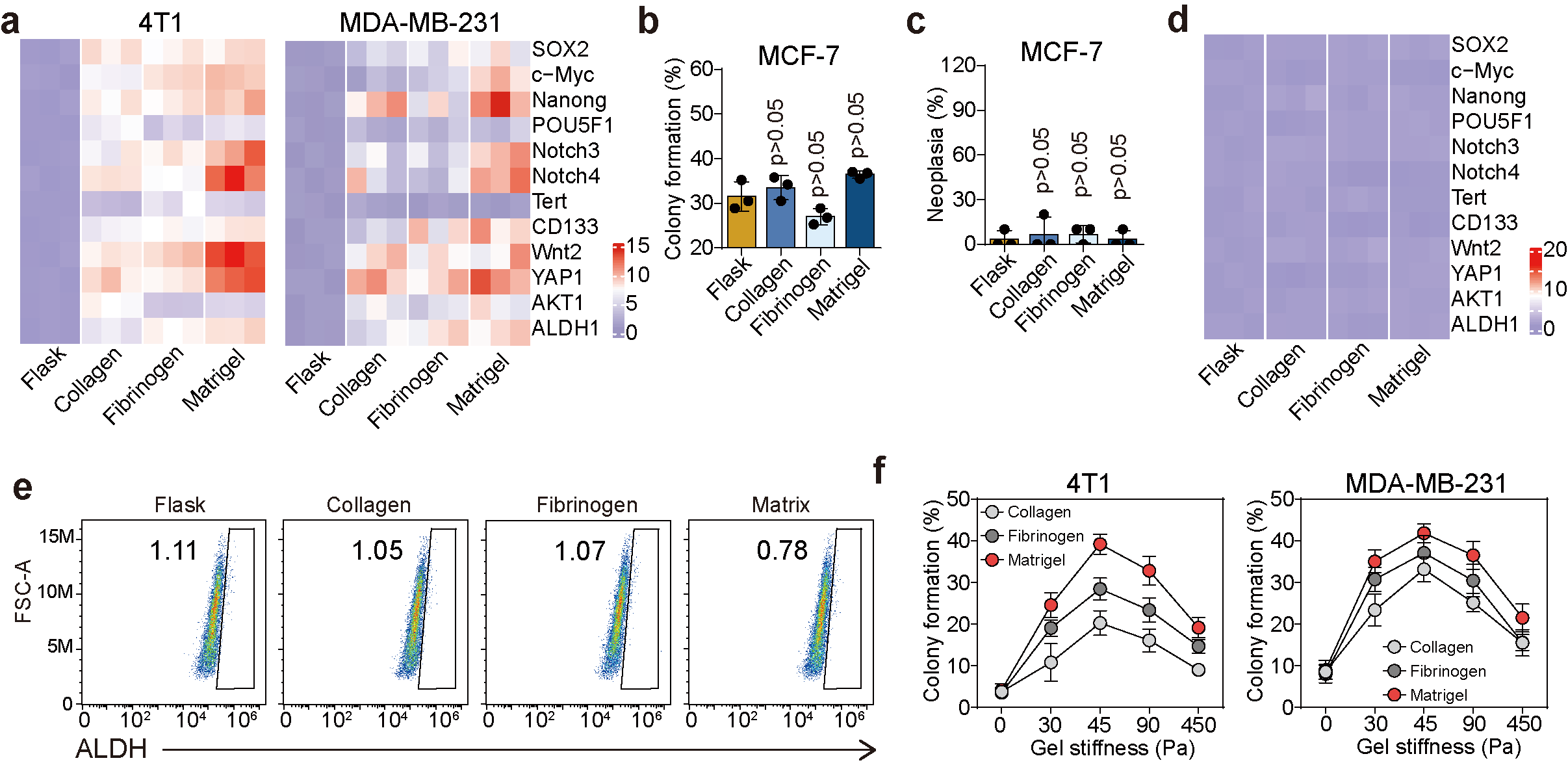
Figure. S1. The ECM-induced biomechanical force promoted breast tumor stemness**. **a,** Heatmap of stem-associated genes (SOX2, c-Myc, Nanong, POU5F1, Notch3, Notch4, Tert, CD133, Wnt2, YAP1, AKT1, ALDH1) expression in 4T1 and MDA-MB-231 cells cultured in flask and different 3D gels (collagen, fibrinogen and Matrigel) for 3 days, determined by qPCR. **b** and **c**, MCF-7 cells were treated with PBS or soluble collagen I, fibrinogen and Matrigel compounds (collagen, laminin, fibronectin) for 3 days. In vitro colony formation (**b**) and in vivo tumor formation assay(n=10) (**c**) was then performed. **d**, Heatmap of stem-associated genes (SOX2, c-Myc, Nanong, POU5F1, Notch3, Notch4, Tert, CD133, Wnt2, YAP1, AKT1, ALDH1) expression in MCF-7 cells treated with PBS or soluble collagen I, fibrinogen and Matrigel compounds (collagen, laminin, fibronectin) for 3 days. **e**, MCF-7 cells were treated with PBS or soluble collagen I, fibrinogen and Matrigel compounds (collagen, laminin, fibronectin) for 3 days. ALDH1+ cell subpopulations were determined by flow cytometry. **f**, 4T1 and MDA-MB-231 cells were seeded in different 3D gels (collagen, fibrinogen and Matrigel) with different stiffness (0, 30, 45, 90 and 450 Pa) for 3 days. Then in vitro colony formation assay was performed. The representative images of tumor cells during AFM analysis were shown. Three independent experiments were performed. Data are represented as mean ± SEM. P<0.05, significantly different. Create a page break and paste in the Figure above the caption.

Figure. S2.


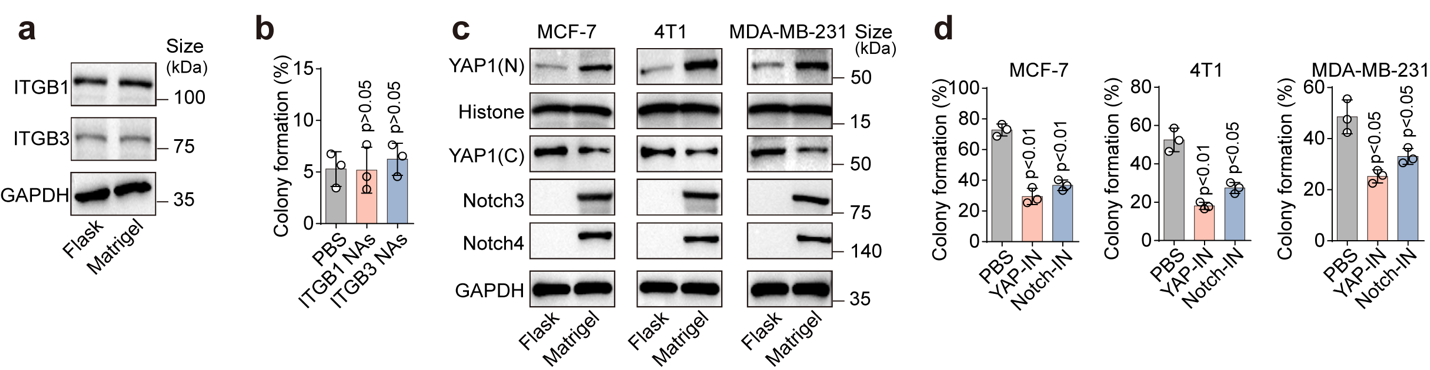
 **Figure. S2. ECM compounds bind to integrins to transduce biomechanical force signals. a**, Western blotting of integrin β1 and β3 in MCF-7 cells treated with PBS or soluble Matrigel compounds (collagen, laminin, fibronectin) for 3 days. **b**, In vitro colony formation of MCF-7 cells treated with PBS or integrin β1- or β3-neutralizing antibodies for 3 days. **c**, Western blotting of YAP1, Notch3 and Notch4 in MCF-7/4T1/MDA-MB-231 cells cultured in flask or 3D Matrigel. **d**, MCF-7/4T1/MDA-MB-231 cells were cultured in 3D Matrigel and treated with YAP-TEAD-IN-1 (50 nM) and Notch inhibitor 1 (25 nM), then in vitro colony formation was determined.. Create a page break and paste in the Figure above the caption. Three independent experiments were performed. Data are represented as mean ± SEM. P<0.05, significantly different.

Figure. S3.

**
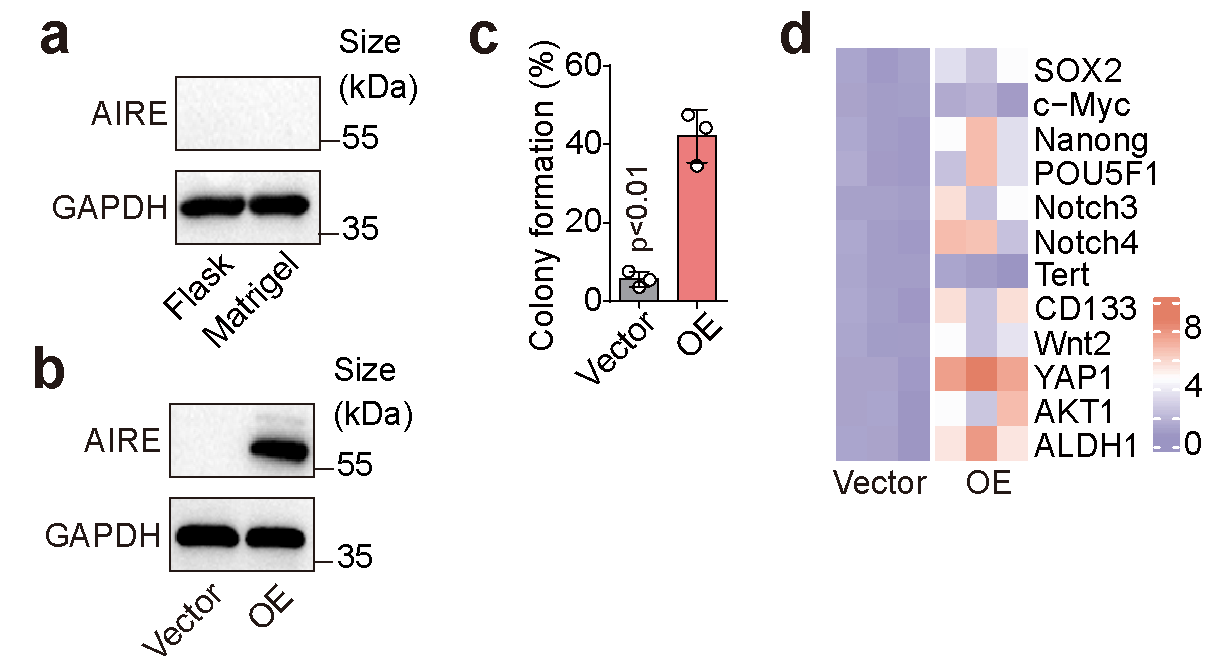
**

**Figure. S3. Integrin-cytoskeleton-AIRE signals are crucial for stemness gene upregulation. a**, Western blotting of AIRE in MCF-7 cells treated with PBS or soluble Matrigel compounds (collagen, laminin, fibronectin) for 3 days. **b**, Western blotting of AIRE in vector and AIRE overexpressed MCF-7cells. **c**, In vitro colony formation of vector and AIRE overexpressed MCF-7 cells. **d**, Heatmap of stemness-associated gene (SOX2, c-Myc, Nanog, POU5F1, Notch3, Notch4, Tert, CD133, Wnt2, YAP1, AKT1, and ALDH1) expression in vector and AIRE overexpressed MCF-7 cells. Three independent experiments were performed. Data are represented as mean ± SEM. P<0.05, significantly different.

Figure S4.

**
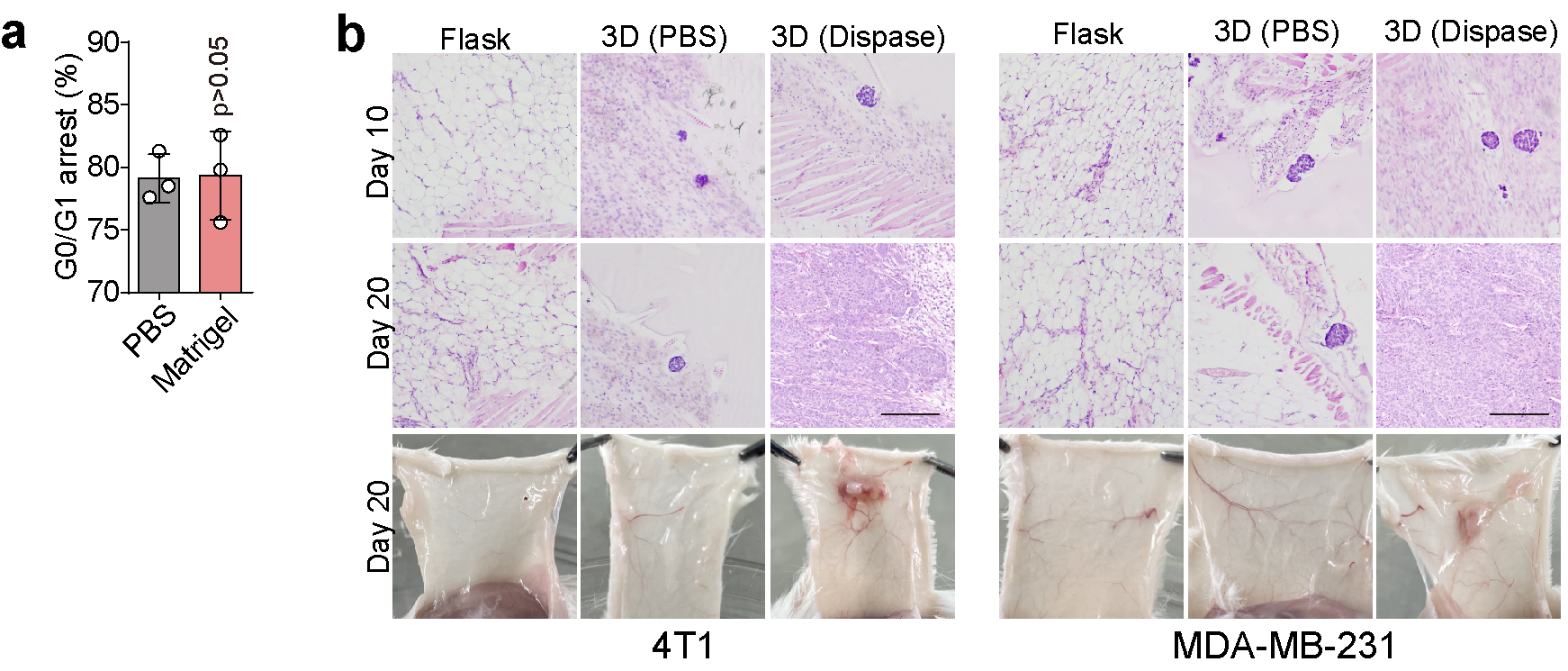
**

**Figure. S4** **ECM-induced biomechanical force drives stem cell-like tumor cell quiescence. a**, Cell cycle analysis of MCF-7 cells treated with PBS or soluble Matrigel compounds (collagen, laminin, fibronectin) for 3 days. **b**, 5×10^3^ MCF-7 or MDA-MB-231 cells were encapsulated in a 450-Pa 3D Matrigel (or not) and subcutaneously implanted into mice(n=10). On days 3 and 5, the mice were treated with PBS or dispase administered via subcutaneous injection. H&E staining of the hypodermis in each group was performed on days 10 and 20. The scale bar was 500 μm. Three independent experiments were performed. Data are represented as mean ± SEM. P<0.05, significantly different.

Figure S5.

**
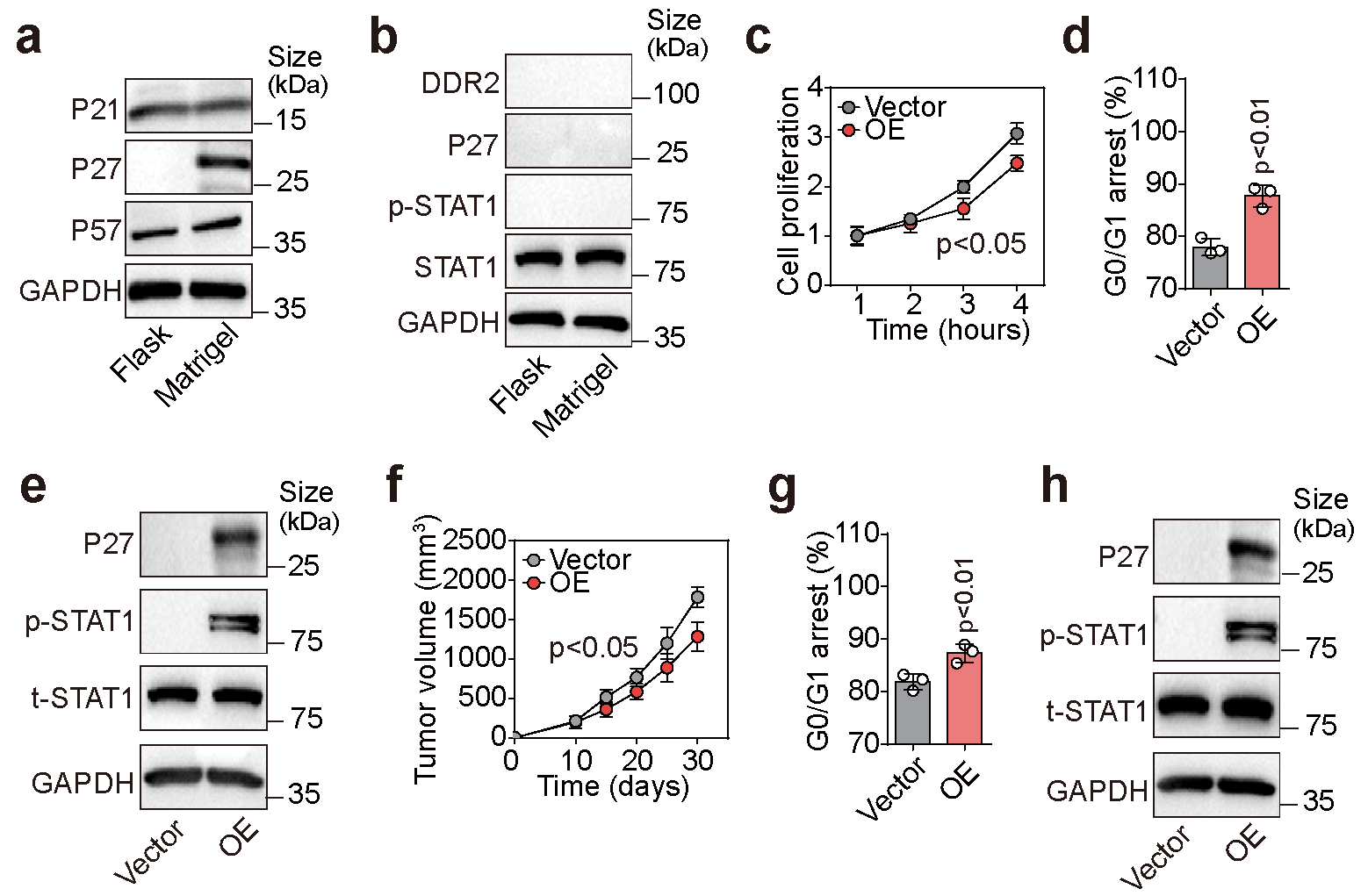
**

**Figure. S5****. The biomechanical force promoted tumor cell quiescence through DDR2 signaling.** **a**, Western blotting of P21, P27 and P57 in MCF-7 cells cultured in flask or 3D Matrigel (450 Pa). **b**, Western blotting of DDR2, phosphorylated STAT1, total STAT1, and P27 in MCF-7 cells cultured with PBS or soluble Matrigel compounds (collagen, laminin, fibronectin) for 3 days. **c**~**e**, DDR2 was overexpressed in MCF-7 cells. Then cell proliferation (**c**), cell cycle (**d**) and P27/STAT1 expression (**e**) were analyzed. **f**~**h**, 1×10^6^ vector or DDR2 overexpressed MCF-7 cells were subcutaneously injected into mice(n=6). The tumor volume was recorded (**f**). Tumor cells were isolated on day15 and cell cycle (**g**) and P27/STAT1 expression (**h**) were examined. Create a page break and paste in the Table above the caption. Three independent experiments were performed. Data are represented as mean ± SEM. P<0.05, significantly different.
